# Resting‐state effective connectivity is systematically linked to reappraisal success of high‐ and low‐intensity negative emotions

**DOI:** 10.1002/hbm.26667

**Published:** 2024-03-27

**Authors:** Carmen Morawetz, Stella Berboth, Stefan Bode

**Affiliations:** ^1^ Department of Psychology University of Innsbruck Innsbruck Austria; ^2^ Department of Psychiatry and Psychotherapy Charité – Universitätsmedizin Berlin Germany; ^3^ Melbourne School of Psychological Sciences The University of Melbourne Melbourne Victoria Australia

**Keywords:** dynamic causal modeling, effective connectivity, emotion regulation, fMRI, reappraisal, resting‐state, ultra‐high field

## Abstract

Emotion regulation is a process by which individuals modulate their emotional responses to cope with different environmental demands, for example, by reappraising the emotional situation. Here, we tested whether effective connectivity of a reappraisal‐related neural network at rest is predictive of successfully regulating high‐ and low‐intensity negative emotions in an emotion‐regulation task. Task‐based and resting‐state functional magnetic resonance imaging (rs‐fMRI) data of 28 participants were collected using ultra‐high magnetic field strength at 7 Tesla during three scanning sessions. We used spectral dynamic causal modeling (spDCM) on the rs‐fMRI data within brain regions modulated by emotion intensity. We found common connectivity patterns for both high‐ and low‐intensity stimuli. Distinctive effective connectivity patterns in relation to low‐intensity stimuli were found from frontal regions connecting to temporal regions. Reappraisal success for high‐intensity stimuli was predicted by additional connections within the vlPFC and from temporal to frontal regions. Connectivity patterns at rest predicting reappraisal success were generally more pronounced for low‐intensity stimuli, suggesting a greater role of stereotyped patterns, potentially reflecting preparedness, when reappraisal was relatively easy to implement. The opposite was true for high‐intensity stimuli, which might require a more flexible recruitment of resources beyond what is reflected in resting state connectivity patterns. Resting‐state effective connectivity emerged as a robust predictor for successful reappraisal, revealing both shared and distinct network dynamics for high‐ and low‐intensity stimuli. These patterns signify specific preparatory states associated with heightened vigilance, attention, self‐awareness, and goal‐directed cognitive processing, particularly during reappraisal for mitigating the emotional impact of external stimuli. Our findings hold potential implications for understanding psychopathological alterations in brain connectivity related to affective disorders.

## Graphical abstract

In our study, we sought to explore a novel dimension of emotion regulation by investigating whether resting state effective connectivity, as measured through ultra‐high field fMRI at 7 Tesla, can serve as a predictive marker for the successful regulation of high‐ and low‐intensity negative emotions.

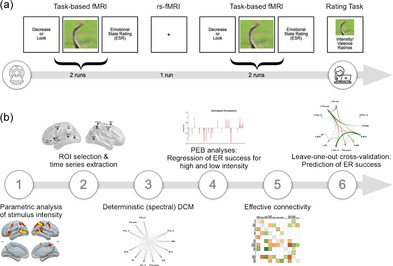

## 1 INTRODUCTION

In our ever‐changing and emotionally demanding environments, the control of our emotional responses is critical for moderating our behaviors (Debrot, 2012; Kobylińska & Kusev, 2019; Levy‐Gigi & Shamay‐Tsoory, 2017; Niven et al., 2015; Sahi et al., 2020), successfully navigating social interactions (Cameron & Overall, 2018; Grommisch et al., 2020; Sahi et al., 2020; Williams et al., 2018), and maintaining our mental well‐being (Newman & Nezlek, 2022; Quoidbach et al., 2010; Rammensee et al., 2023). Different situations are often suited for the use of specific regulation strategies (Matthews et al., 2021; Sheppes, 2020), but aspects of the stimuli that trigger the emotional response, such as their intensity, will also determine how successful emotion regulation will be in a given situation (Aldao, 2013; Aldao & Tull, 2015; Dore et al., 2016). People further differ in their ability/capacity to regulate their emotions, and some mental disorders, such as depression, anxiety, and borderline personality disorder, are explicitly characterized by difficulties in effectively regulating one's emotions. Contextual factors are also important predictors of the vulnerability to these mental disorders (Aldao et al., 2016; Kring & Sloan, 2010; Sloan et al., 2017). However, healthy individuals also have substantial differences in their emotion regulation capacity. This fact is further reflected in a growing body of work investigating training programs aimed at helping people to improve their emotion regulation skills in general and in specifically challenging situations (e.g., Barkus, 2020; Gatto et al., 2022; LeBlanc et al., 2020; Linhartová et al., 2019; Liu et al., 2020; Pan et al., 2022; Ungar & Theron, 2020). Despite substantial progress in understanding the general cognitive and neural mechanisms underlying emotion regulation (e.g., Buhle et al., 2014; Kohn et al., 2014; Morawetz et al., 2020; Morawetz, Bode, Baudewig, & Heekeren, 2017), the neurocognitive origins of these individual and situational differences in emotion regulation capacity remain poorly understood.

One intriguing possibility is that individual differences in emotion regulation capacity are related to the dynamic organization of the emotion‐regulation brain network at rest. Recent research has indeed shown that the strength of the expression of behavioral effects of interest, both in the cognitive and affective domain, was associated with differences in resting‐state effective connectivity (Jamieson et al., 2021; Morawetz et al., 2022; Voigt et al., 2020). These studies have employed spectral dynamic causal modeling (spDCM) to resting‐state functional magnetic resonance imaging (rs‐fMRI) data to identify causal connections between distributed brain areas (Friston et al., 2014; Park et al., 2018; Razi et al., 2015, 2017) to predict individual differences in the behavioral effects of interest.

So far, two fMRI studies examined the effective connectivity, that is, the causal directed connectivity, between brain regions during active emotion regulation. These studies provided evidence for a prefrontal feedback mechanism between the dorsolateral prefrontal cortex (dlPFC) and ventrolateral prefrontal cortex (vlPFC) during emotion regulation (Morawetz, Bode, Baudewig, Jacobs, & Heekeren, 2016), and a fronto‐amygdala network in which ventromedial prefrontal cortex (vmPFC) to amygdala and amygdala to pre‐supplementary motor area (SMA) connectivity was modulated by reappraisal (Steward et al., 2021). Moreover, research utilizing transcranial direct current or magnetic stimulation (tDCS or TMS) underscores the significance of the dlPFC, vlPFC, and temporoparietal junction in the process of reappraisal in a causal manner (Feeser et al., 2014; He et al., 2023; Lantrip et al., 2017; Powers et al., 2020; Pripfl & Lamm, 2015). Combining TMS with causal effective connectivity analysis revealed that TMS‐induced vlPFC facilitation is related to increased activity in the prefrontal cortex (vlPFC and vmPFC) and attenuated activity in subcortical regions including the amygdala and insula (He et al., 2023). There is also work linking activation in brain regions more directly to emotion regulation success, in particular for prefrontal regions, including vlPFC, vmPFC, dlPFC, and the SMA (Banks et al., 2007; Diekhof et al., 2011; Lee et al., 2012; Morawetz, Bode, Baudewig, Kirilina, & Heekeren, 2016; Wager et al., 2008). These studies provide first insights into the mechanisms by which cortical and subcortical regions interact to down‐regulate emotional responses. The results are compatible with top‐down models, which assume that frontal and parietal cognitive control regions inhibit the activity in emotion‐generation regions such as the amygdala (Berboth & Morawetz, 2021; Buhle et al., 2014; Ochsner et al., 2012; Wager et al., 2008).

The second unexplored factor in this context is whether neural network dynamics might differ in relation to the intensity of the stimulus that triggers the negative emotion to be regulated. Evidence for the importance of stimulus intensity comes from studies showing that participants prefer to choose different regulation strategies when confronted with high‐ or low‐intensity stimuli (Rammensee et al., 2023; Sheppes, 2020). Other studies reported that stimulus intensity modulated components of the event‐related potential (ERP) when participants used reappraisal as an emotion regulation strategy. Stronger amplitudes were found for the centro‐parietal positivity (CPP) and the late positive potential (LPP) in response to high‐intensity stimuli as compared with low‐intensity stimuli (Shafir et al., 2015, 2016). Further, two fMRI studies (Moodie et al., 2020; Silvers et al., 2015) have shown that emotion regulation via reappraisal was generally related to increased activity in dorsomedial prefrontal cortex (dmPFC) and left lateral PFC regions. For high‐intensity stimuli (but not low‐intensity stimuli), one study found additional recruitment of the left dlPFC, the right lateral PFC, and the anterior portion of the dmPFC during emotion regulation (Silvers et al., 2015). The other study reported additionally increased activity in response to high‐intensity stimuli in the left vlPFC, the right anteromedial temporal cortex and right dmPFC (Moodie et al., 2020). Together, these findings support the idea that regulating high‐intensity emotional responses requires greater cognitive resources.

In summary, earlier fMRI investigations and research on transcranial stimulation emphasize the crucial involvement of prefrontal regions in successful reappraisal. Additionally, contextual factors, including stimulus intensity, have been identified as modulators of prefrontal activity, leading to a higher demand for cognitive resources. Therefore, effective reappraisal appears to rely on both prefrontal activity and the intensity of the reinterpreted stimulus. However, the potential association between resting‐state effective connectivity among regions implicated in reappraisal, the success of emotion regulation, and stimulus intensity remains unexplored. No study has examined whether individuals' proficiency in mobilizing cognitive resources to reappraise high‐intensity stimuli already manifests in their resting‐state effective connectivity profile within the reappraisal network.

**FIGURE 1:**
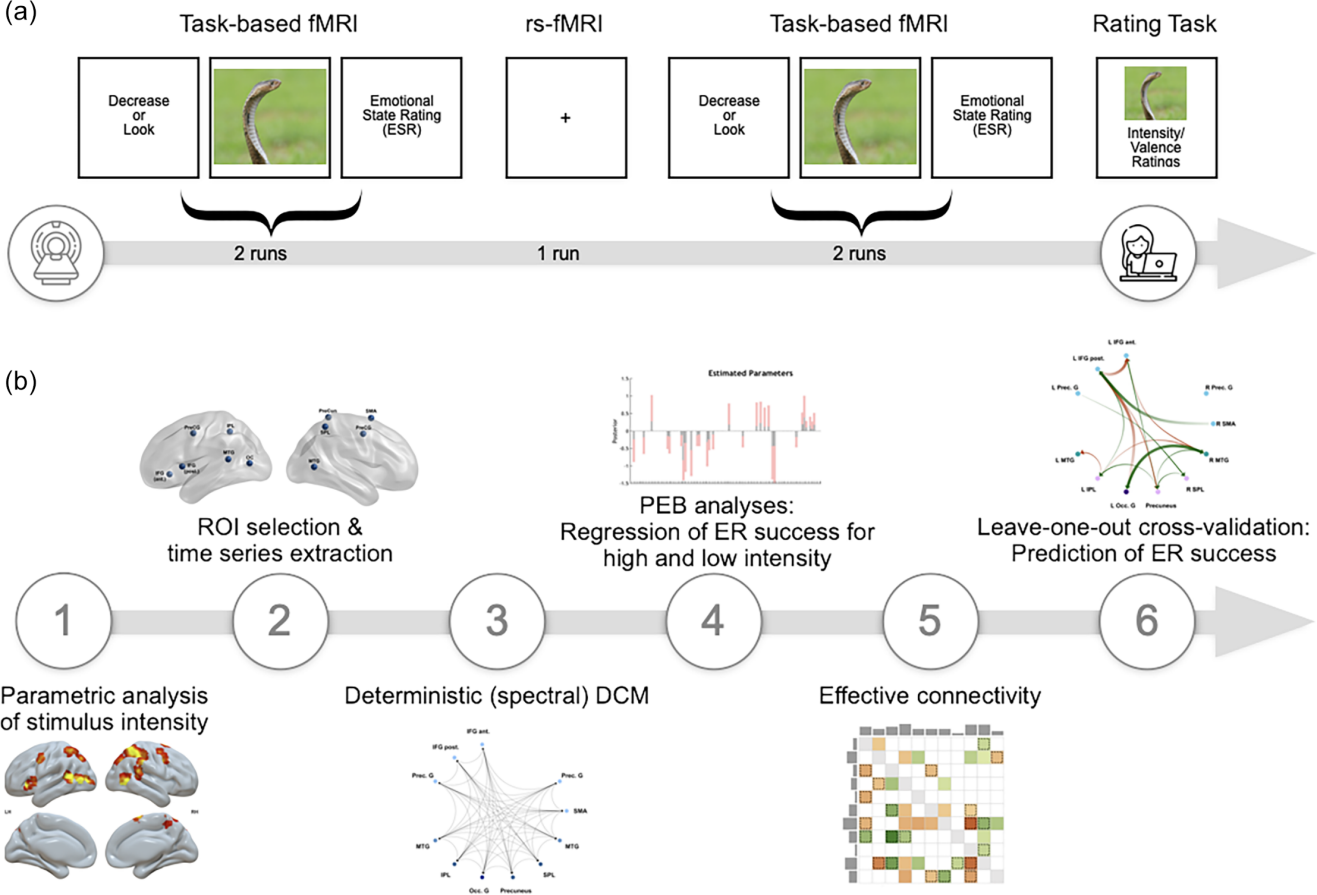
Overview of key processing steps for predictive analysis of reappraisal success from rs‐fMRI spectral DCM parameters from regions parametrically modulated by stimulus intensity. (a) Sequence of tasks performed inside and outside the scanner. Inside the scanner, participants performed a standard emotion regulation task and were instructed to either down‐regulate their emotions using reappraisal (Decrease) or to look at the images attentively without regulating their emotions (Look). After the regulation phase, participants indicated their current emotional state using a rating scale. The task‐based fMRI was interleaved by one rs‐fMRI run. A picture rating task was performed outside the scanner. All images were rated on valence from 1 (very negative) to 9 (very positive) and on intensity from 1 (calm) to 9 (very intense). (b) Step 1: The intensity ratings (collected at (a)) were used to identify regions of interest (ROIs) that were parametrically modulated by intensity during reappraisal. Step 2: For the rs‐fMRI analysis, time series were extracted from the task‐based ROIs (determined in Step 1). Steps 3, 4, and 5: Using deterministic spectral DCM and the hierarchical Parametric Empirical (PEB) framework, the interaction between connectivity changes and ER success in response to high‐ and low‐intensity stimuli were investigated. Step 6: ER success was predicted by rs‐fMRI effective connectivity using leave‐one‐out cross‐validation.

The aim of the present study was to examine whether individual differences in reappraisal success are related to the dynamic organization of the reappraisal‐related brain network at rest. Unlike previous studies, we used ultra‐high field fMRI at 7 Tesla (T), which has an advanced spatial resolution, making it possible to image even small brain structures with high precision. We also conducted a multisession experiment, meaning that we collected more data than any previous study, increasing the precision of our analyses. We investigated our research question for high and low stimulus intensity separately to account for the possibility that different effective connectivity profiles might underlie emotion regulation for these different stimulus classes. First, during a validated reappraisal emotion regulation task, brain regions parametrically modulated by stimulus intensity were identified (Figure 1). Next, resting state‐fMRI (rs‐fMRI) was used to measure low‐frequency fluctuations in the blood oxygen level‐dependent (BOLD) signal. In separate analyses for low‐ and high‐intensity stimuli, we then applied spDCM to the resting state data to predict individuals' overall reappraisal success in the emotion regulation task.

In line with compelling evidence that supports the notion that resting‐state connectivity might be related to cognitive task activations and forecast brain activity during task execution (Cole et al., 2016, 2021; Smith et al., 2009; Tavor et al., 2016), we hypothesized that changes in effective connectivity at rest in the network of brain regions related to reappraisal would already be predictive for future reappraisal success (Morawetz et al., 2022). Given the absence of prior studies examining effective connectivity in the resting‐state, we did not have directed hypotheses but had to take an exploratory approach. Thus, we hypothesized that different connectivity patterns might emerge depending on stimulus intensity. We did, however, hypothesize that specific connections within the neural network that demonstrate predictability for reappraisal success were highly reliable, which we tested directly by employing Leave‐One‐Out Cross‐Validation (LOOCV).

## 2 MATERIALS AND METHODS

### 2.1 Participants

A total of 32 participants were recruited for the fMRI experiment (27 females, mean age = 22.56 years, *SD* = 3.06, range = 19–35). Using a standard MRI screening questionnaire, it was established that participants were right‐handed and had no history of neurological or psychiatric disorders. All participants gave written, informed consent to participate and reported no history of neurological or psychiatric disorders and were compensated with 60€ for participation.

Four participants were excluded from the study due to excessive head movement (movement >3 mm/>3 degrees in one direction). The final sample therefore consisted of 28 right‐handed, healthy participants with normal or corrected to normal vision (23 females, 5 males; mean age = 22.75 years, SD = 3.17, range = 19–35).

The data collection consisted of three fMRI sessions separated by ~1 week. Each session included the emotion regulation task in the scanner, assessment of the resting‐state fMRI data, and a behavioral rating task outside the scanner after the fMRI data collection (Figure 1). Behavioral intensity ratings of stimuli (behavioral rating task) were missing due to technical problems for *n* = 7 participants in session 1 and *n* = 3 participants in session 2. As those behavioral ratings were essential for our task‐based fMRI analyses, for *n* = 3 participants only session 3 and *n* = 4 participants only session 2 and 3 were included in the subsequent task‐based fMRI analyses, that is, the parametric modulation of emotion regulation.

The study was approved by the local ethics committee of the Medical University of Vienna (1309/2018) and carried out in accordance with the Declaration of Helsinki.

### 2.2 Experimental design

#### 2.2.1 Procedure

Participants underwent three fMRI sessions separated by ~1 week. Each fMRI session involved a resting‐state fMRI run and four runs of an emotion regulation task and was followed by a behavioral rating experiment. This resulted in three resting‐state fMRI runs and 12 runs of the emotion regulation task in total. In the behavioral rating experiment outside of the scanner, participants were instructed to rate the stimuli of the preceded emotion regulation task on valence and arousal (i.e., intensity), and the latter was used as key variable for this research. The key processing steps of the behavioral and imaging data are outlined in Figure 1.

#### 2.2.2 Stimuli and stimulus rating task

Stimuli consisted of 240 aversive images in total, 81 of which were from the International Affective Picture System (IAPS; mean arousal/intensity = 6.17, SD = 0.62; mean valence = 2.26, SD = 0.59) (Bradley & Lang, 2007) and 159 were taken from the Nencki Affective Picture System (NAPS; mean arousal/intensity = 6.42, SD = 0.63; mean valence = 3.13, SD = 0.64) (Marchewka et al., 2014). Valence and arousal/intensity values were based on the normative ratings, which are available on a Likert scale from 1 (very negative/very calm) to 9 (very positive/very intense). We included high‐ and low‐intensity images evenly distributed across all three sessions and runs to elicit emotional responses. The stimulus set was divided into 24 sets of images matched in content, valence, and intensity across the two task conditions (*Look* or *Decrease*, see below) and three sessions, each consisting of four runs, to ensure that emotion induction was comparable.

After each of the three fMRI sessions, our participants also rated the presented images on both valence and intensity on a nine‐point Likert scale from 1 (very positive/calm) to 9 (very negative/highly intense) using the Self‐Assessment Manikin (Bradley & Lang, 2007) (Figure 1a). These stimulus ratings were used in the task‐based fMRI analyses as parametric regressors. Further, based on these individual ratings the stimuli were categorized into “high‐intensity” and “low‐intensity” stimuli for each participant.

During the fMRI experiment, images were presented in the center of the screen with an 800 × 600 pixels display using the stimulation software Presentation (Version 20.2, Neurobehavioral Systems, USA).

#### 2.2.3 Emotion regulation task

A well‐established emotion regulation task was used, adapted from previous studies (Morawetz, Bode, Baudewig, Jacobs, & Heekeren, 2016; Morawetz, Bode, Baudewig, Kirilina, & Heekeren, 2016; Ochsner et al., 2004) (Figure 1b). Two conditions were implemented in the experiment. In the *Look* condition, participants were asked to view the images attentively and allow themselves to experience/feel any emotional responses that these might elicit without trying to influence or change them. In the *Decrease* condition, participants were asked to reappraise the negative emotional value of the images to reduce the intensity of the negative emotion. Participants were instructed to reduce the negative impact of the depicted situation by distancing themselves from the image by becoming a detached observer, taking the perspective of a professional observer, or thinking that the depicted situation is not real. Before the first fMRI measurement, participants received a training session of the experiment described below to practice the reappraisal strategy. The training session included four trials of each condition (*Look* and *Decrease*) resulting in eight trials in sum.

Pictures were presented in an event‐related design. Each trial started with the instruction cue (2 s) showing either the words “Down‐regulate” (*Decrease*) or “Maintain” (*Look*) at the center of the screen. Subsequently, a fixation cross was presented (2–6 s jitter), followed by the aversive image (8 s). After the image, another fixation cross (2–6 s jitter) was presented, followed by a rating of the current emotional state (4 s) (continuous scale from very negative to very positive; −200 to +200). Finally, a fixation cross (2–6 s jitter) concluded the trial. One experimental run consisted of 20 trials (10 per intensity condition) that were randomized within each run. Each experimental session consisted of four runs resulting in 80 trials per session and a total of 240 trials for all three sessions.

#### 2.2.4 fMRI data acquisition

Whole‐brain functional and anatomical images were acquired using a SIEMENS Magnetom ultra high‐field 7.0 Tesla MR scanner and a 32‐channel head coil. A high‐resolution 3D T1‐weighted dataset was acquired for each participant and each session (224 sagittal sections, 0.7 × 0.7 × 0.7 mm^3^; 224 × 224 data acquisition matrix).

Participants were instructed to rest with open eyes while fixating a central cross‐hair for the task‐free rs‐fMRI scan. Functional images were acquired using the CMRR multiband EPI sequence (TR = 1.4 s; TE = 23 ms; 78 slices; voxel size = 1.5 × 1.5 × 1.0 mm^3^; 0.2 mm slice gap; field of view = 192 × 192 × 97.5 mm^3^; flip angle = 62°). During a total acquisition time of 6 min, 258 whole‐brain volumes were recorded in each session. For the data analyses, resting‐state fMRI data of all three sessions were concatenated, resulting in an acquisition time of 18 min in total. Resting‐state fMRI sessions in which participants showed extensive head movement (>3 mm/>3 degrees in one direction) were excluded from further analyses. This resulted in excluding eight resting‐state sessions (two resting‐state fMRI sessions of three participants; one resting‐state fMRI session of two participants).

During the task‐based fMRI runs, functional images were also acquired using the CMRR multiband EPI sequence (TR = 1.4 s; TE = 23 ms; 78 slices; voxel size = 1.5 × 1.5 × 1.0 mm^3^; 0.2 mm slice gap; field of view = 192 × 192 × 97.5 mm^3^; flip angle = 62°). For each task‐based experimental run, 371 whole‐brain volumes were recorded.

### 2.3 Behavioral data analyses

Behavioral data included emotional state ratings for each trial collected during the fMRI experiment (i.e., Emotion Regulation Task) and valence and intensity ratings collected after each fMRI session (i.e., Stimulus Rating Task). In addition to the behavioral data analyses, all subjective ratings were used as variables of interest in the task‐based fMRI analyses, that is, whole‐brain parametric regression analyses to identify brain regions modulated by intensity, valence, and emotional state during the emotion regulation task.

#### 2.3.1 Intensity ratings

To classify trials of the Emotion Regulation Task into high‐ or low‐intensity trials, a median split of the intensity ratings for all stimuli/trials retrieved from the Stimuli Rating Task was conducted for each participant and each session. This classification in “high‐intensity” and “low‐intensity” stimuli was used for the subsequent analyses.

#### 2.3.2 Emotional state ratings and reappraisal success

First, we performed a repeated‐measures ANOVA with the within‐subject factors “regulation” (*Look*, *Decrease*) and “intensity” (*high‐intensity*, *low‐intensity*) to analyze the effects of these variables on the emotional state ratings, followed by post hoc paired *t*‐tests (two‐tailed; *p*‐values Bonferroni‐corrected).

Second, reappraisal success scores were calculated using the emotional state ratings acquired after each trial. Each trial was categorized as either successful/unsuccessful by subtracting the mean of the *Look* trials from the emotional state rating on each trial of the *Decrease* condition within each run. Hence, positive values represent successful trials (participant reported weaker negative affect during *Decrease* compared with the *Look* condition), while negative values represent unsuccessful trials (participant reported stronger negative affect compared with the *Look* condition) (Berboth et al., 2021; Morawetz et al., 2018, 2021; Morawetz, Bode, Baudewig, & Heekeren, 2017; Morawetz, Bode, Baudewig, Kirilina, & Heekeren, 2016). We then calculated the percentage of successfully regulated trials to determine how successful participants regulated their emotions for low‐ and high‐intensity stimuli, respectively. For this, the number of successfully regulated trials was divided by the number of all *Decrease* trials for each stimulus intensity separately (positive difference score). These percentage scores indicate how many trials were successful in relation to all *individually* defined *high‐* and *low‐intensity* regulation trials (Morawetz, Bode, Baudewig, Kirilina, & Heekeren, 2016; Morawetz, Bode, Derntl, & Heekeren, 2017; Morawetz, Kellermann, Kogler, Radke, et al., 2016). We tested for the effect of intensity on success scores by performing a paired *t*‐test (two‐tailed). These individual reappraisal success scores for high‐ and low‐intensity stimuli, respectively, were also used as covariates in the spectral dynamic causal modelling (spDCM) analyses (see below).

Third, we determined the relationship between stimulus intensity ratings and emotional state ratings using Pearson correlations for each regulation condition separately.

### 2.4 fMRI data analyses

#### 2.4.1 Preprocessing

Functional imaging data were analyzed using SPM12 (Statistical Parametric Mapping, Wellcome Centre for Human Neuroimaging, London, UK) in Matlab R2018b (MathWorks, Natick, MA). The standard preprocessing steps consisted of realignment, slice‐time correction, and coregistration to the respective structural image of the participant. The structural images were then segmented and EPIs were transformed into the stereotactic normalized standard space (Montreal Neurological Institute, MNI) by using the segmentation transformation matrix. Finally, data was smoothed with an isotropic Gaussian kernel (6 mm full‐width at half‐maximum). The resting state data of the three sessions were concatenated including session as a regressor at the first level (Cho et al., 2021).

#### 2.4.2 Whole‐brain group level parametric analyses and time series extraction

First, whole‐brain parametric analyses of the task‐based fMRI data were conducted. The parametric modulation by stimulus intensity was then used to determine the Regions of Interest (ROIs) for the subsequent spDCM analyses (Figure 1b).

The first‐level general linear model (GLM) consisted of stimulus onset vectors representing the instruction (duration 2 s), the emotion regulation phase during stimulus viewing split by regulation conditions (*Look*, *Decrease*) (duration 8 s), and the rating phase (4 s) that were each convolved with the hemodynamic response function. Six movement parameters were included in the model as nuisance regressors. To identify brain regions that were associated with reappraisal and modulated by stimulus intensity, individual intensity ratings for each stimulus were used as a parametric regressor for the *Look* and *Decrease* condition, respectively. Contrast images of the parametrically modulated brain activation during the *Decrease* and *Look* condition were calculated for each participant and each session.

The second‐level group analysis was based on random‐effects analyses of the single‐subject contrast images to examine how trial‐by‐trial variability in (a) stimulus intensity, (b) stimulus valence, and (c) emotional state ratings modulated neural activation during the emotion regulation task. This resulted in six separate whole‐brain parametric regressions analyses: stimulus intensity during (1) the *Decrease* condition and (2) the *Look* condition, stimulus valence during (3) the *Decrease* condition, and (4) the *Look* condition, and emotional state ratings during (5) the *Decrease* condition, and (6) the *Look* condition (Figure S3). To account for the effect of session, session was included as a factor in each analysis.

For the present analysis, we were specifically interested in the modulation effects by stimulus intensity. To identify the ROIs for the subsequent spDCM analysis, the parametric regression contrast of stimulus intensity during the *Decrease* condition was exclusively masked by the Look condition. This allowed us to determine brain regions that were parametrically modulated by stimulus intensity *only* during reappraisal (Figure 1b). The *t*‐statistics were thresholded at an initial cluster‐defining threshold of *p* < .001 and a relatively liberal threshold at the cluster level of *p* < .05. Masking was thresholded at *p* < .05. Coordinates of the results are reported in MNI space. This parametric analysis yielded 11 ROIs (Figure 1c, Table 1).

**TABLE 1:** Results of the parametric modulation of intensity ratings during the decrease condition masked by the parametric modulation effect of intensity ratings during the Look condition.

Region	Side	Coordinates			Cluster size	*t* value	*p* value
		*x*	*y*	*z*			
Middle temporal gyrus (MTG)	R	51	−66	6	588	5.63	.002
Superior parietal lobe (SPL)	R	33	52	57	4489	5.49	<.001
Middle temporal gyrus (MTG)	L	−51	−46	13	613	5.00	.002
Occipital gyrus (Occ. G)	L	−48	−73	8	386	4.84	.009
Supplementary motor area (SMA)	R	12	6	68	202	4.72	.047
Inferior frontal gyrus ant. (IFG ant.)	L	−32	28	−6	294	4.65	.019
Precentral gyrus (Prec. G)	R	33	−4	48	956	4.53	<.001
Precentral gyrus (Prec. G)	L	−34	−2	46	298	4.50	.019
Inferior parietal lobe (IPL)	L	−24	−48	48	1237	4.48	<.001
Inferior frontal gyrus post. (IFG post.)	L	−46	12	4	310	4.10	.017
Precuneus		2	−48	69	252	3.89	.029

*Note*: Coordinates refer to MNI coordinate system. *p* < .05 cluster‐wise uncorrected (initial cluster‐defining threshold *p* < .001). Uncorrected mask *p*‐value *p* = 0.05.

Abbreviations: ant., anterior; L, left hemisphere; post., posterior; R, right hemisphere.

After defining the ROIs, preprocessed resting‐state data were used to establish the residuals of a GLM to extract the BOLD fMRI time series. Six head motion parameters and WMF/CSF signals were added to the GLM as nuisance regressors. Time series from each of the aforementioned ROIs were extracted using the principal eigenvariate of all voxels in a sphere centering at the coordinates in Table 1 with a radius of 6 mm.

#### 2.4.3 Spectral dynamic causal modelling

To estimate the effective connectivity between the 11 ROIs, spDCM was performed using DCM12 as implemented in SPM12. Connectivity between ROIs is measured in Hertz (Hz), with positive values indicating excitatory connections, while negative values represent inhibitory connections. In this analysis, we focused on the modulation of changes in effective connectivity by reappraisal success for high‐ and low‐intensity stimuli, respectively.

At the first level, a fully‐connected model was constructed for each participant (Figure 1c). Next, each participant's specified DCMs were inverted using spectral DCM, which fits the complex cross‐spectral density using a parameterized power‐law model of endogenous neuronal fluctuations (Razi et al., 2015). This results in measures of causal interactions between regions and the amplitude of endogenous neuronal fluctuations within each region. Further, Bayesian Model Reduction (BMR) was used to find the best model to explain the data for each participant (Friston & Penny, 2011). First‐level DCM model convergence statistics, for example, explained variance for each individual's final reduced model, were determined using model fit (spm_dcm_fmri_check.m in SPM12) as a diagnostic tool. Results are reported and illustrated in Figures S4 and S5.

To investigate how the connectivity between the ROIs was modulated by reappraisal success for high‐ and low‐intensity stimuli, respectively, hierarchical models over the parameters were specified within a hierarchical Parametric Empirical framework (PEB, Parametric Empirical Bayes) for DCM (Figure 1c). To evaluate how regions in each network of interest interact, we used Bayesian model comparison to examine the space of possible models. Each model assumed that a different combination of the connectivity parameters could characterize all the participants. Candidate models were obtained by removing one or more connections to produce nested or reduced versions of the full model. The parameters of reduced PEB models can be derived analytically from the full model using BMR. This algorithm implements a greedy search across all the permutations of a small set of parameters whose removal produces the smallest reduction (i.e., greatest increase) in model fit (Friston & Penny, 2011). After Bayesian Model Reduction, the best 256 reduced models from this search procedure were averaged and weighted by their model evidence using Bayesian Model Averaging (BMA). Because the intrinsic self‐connections cannot be pruned, the self‐connections were not interpreted.

Two PEB analyses were conducted using reappraisal success of either high‐intensity or low‐intensity stimuli as predictor for the effective connectivity of the neural network of interest. Reappraisal success scores were modelled as the main regressor of interest and mean‐centered to ensure that the intercept of each model was interpretable as the mean connectivity. Only effects (i.e., changes in effective connectivity) that showed a posterior probability >.95 are reported.

In a final step, leave‐one‐out cross‐validation (LOOCV) was performed to determine the robustness of observed effect sizes (Figure 1c). This procedure assessed the predictive validity of reappraisal success, that is, whether the effect size was large enough to predict a left‐out participant's reappraisal success score from the connectivity within the neural network of interest. LOOCV was performed for each significant connection separately.

## 3 RESULTS

### 3.1 Behavioral results

#### 3.1.1 Emotional state ratings

Testing for the effect of stimulus intensity (high‐ vs. low‐intensity) and emotion regulation condition (Look vs. Decrease) on emotional state ratings by conducting a repeated‐measures ANOVA, revealed a significant main effect of emotion regulation (*F*(1,27) = 52.59, *p* < .001), a significant main effect of intensity (*F*(1,27) = 272.71, *p* < .001) and a significant interaction effect between emotion regulation and intensity (*F*(1,27) = 27.72, *p* < .001) (Figure S1A). Post hoc paired *t*‐tests showed that participants felt significantly less negative after *Decrease* compared with the *Look* condition in response to high‐ and low‐intensity stimuli and less negative in response to low‐ in comparison to high‐intensity stimuli in the *Decrease* and the *Look* condition (Table 2).

**TABLE 2:** Post hoc paired *t*‐tests of the effect of regulation condition and stimulus intensity on emotional state ratings.

Condition	*M* (SD)	(1)			(2)			(3)		
		*t* (27)	*p*	*d*	*t* (27)	*p*	*d*	*t* (27)	*p*	*d*
(1) Decrease_high_	−43.8 (35.5)									
(2) Decrease_low_	−6.47 (33.5)	−12.11	**<.001**	2.29						
(3) Look_high_	−103 (31.2)	7.66	**<.001**	1.45	12.0	**<.001**	2.26			
(4) Look_low_	−46.6 (25.3)	0.41	1	0.08	6.3	**<.001**	1.19	−15.42	**<.001**	2.91

*Note*: The effect size is indicated by Cohen's *d*. Results are Bonferroni corrected for multiple comparisons (*n* = 6). Significant results are indicated in bold.

#### 3.1.2 Regulation success scores

To investigate the effect of stimulus intensity on regulation success scores, a paired *t*‐test (two‐tailed) was conducted, indicating a significant effect of the intensity condition (*t*(27) = −8.98, *p* < .001, Cohen's *d* = −1.7) with success scores being significantly lower for *high‐intensity* stimuli (*M* = 63%, SD = 20%) compared with *low‐intensity* stimuli (*M* = 87%, SD = 11%) (Figure S1B).

**FIGURE 2:**
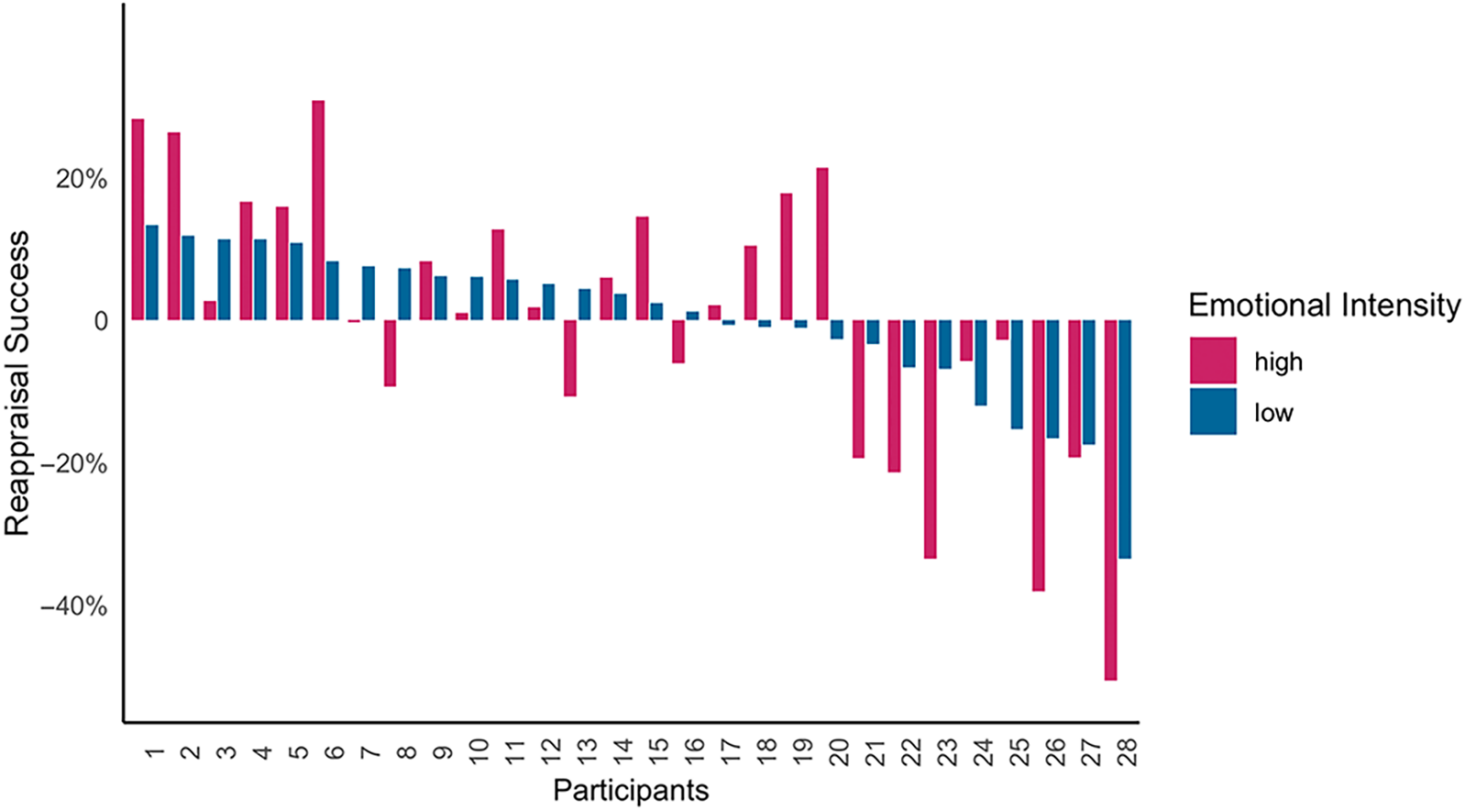
Reappraisal success in response to high‐intensity (purple) and low‐intensity (blue) stimuli for each participant. Success scores were mean‐centered to enable that the intercept of each model was interpretable as the mean connectivity.

Thus, participants were significantly more successful in regulating their negative emotions in response to stimuli that they individually rated as low‐intensity stimuli compared with stimuli rated as high‐intensity stimuli. Notably, regulation success scores for high‐ and low‐intensity stimuli varied considerably across individuals indicating individual differences in the ability to regulate emotions (Figures 2 and S2), which was a precondition for our resting‐state effective connectivity analysis in relation to intensity (see below).

#### 3.1.3 Stimulus intensity and emotional state ratings

Finally, as a sanity check, we tested the relationship between stimulus intensity and emotional state ratings. A correlation analysis, based on the mean intensity ratings and mean emotional state ratings for each stimulus across participants, revealed that stimuli that were rated as high‐intensity stimuli were associated with more negative emotional state ratings in the emotion regulation task (*r* = −.63, *p* < .001) (Figure S1C).

### 3.2 fMRI results

#### 3.2.1 Whole‐brain parametric modulation and selection of ROIs

In a first step, a series of whole‐brain parametric modulation analyses of the task‐based fMRI data was conducted. For this, we first modeled the subjective stimulus intensity ratings given outside the scanner in the stimulus rating task as the parametric regressor for each trial. In a second analysis, we used the valence ratings of each stimulus retrieved from the stimulus rating task as the parametric regressor. In a final analysis, we modeled the emotional state ratings collected during the emotion regulation task as the parametric regressor (Figure S3). The latter two analyses mainly served as sanity checks, as these variables are naturally correlated.

Increased activity in a number of cortical and subcortical regions was positively correlated with stimulus intensity, and negatively correlated with valence and emotional state ratings during *Decrease* and *Look* (see Tables S1, S2, and S3 for details). In other words, increased activity within these regions was related to increased stimulus intensity, decreased valence, and decreased emotional state ratings (i.e., more negative feelings). Specifically, regions involved in emotion generation, such as the amygdalae as well as regions involved in emotion regulation, including the middle temporal gyrus (MTG), supramarginal gyrus (SMG), inferior parietal lobe (IPL), and precentral gyrus were modulated on a trial‐by‐trial basis by stimulus intensity during reappraisal. Stimulus valence correlated negatively with activity in the fusiform gyri, IPL, superior parietal lobe (SPL), IFG, and medial frontal gyrus during decreasing emotional responses. The emotional state ratings were related to activity in the IFG, SPL, MTG, SMG, and caudate such that less negative ratings were related to decreased activity during reappraisal.

**FIGURE 3:**
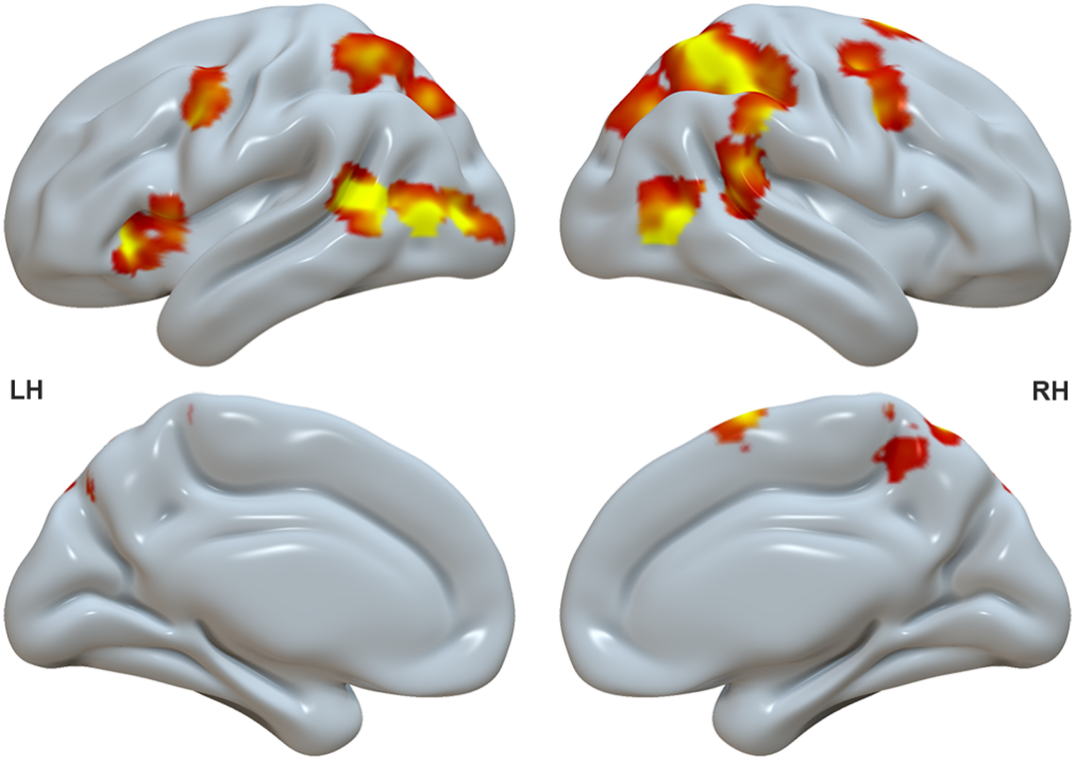
Brain regions that were parametrically modulated by trial‐by‐trial variability in stimulus intensity during reappraisal masked by the control condition (FWE *p* < .05 uncorrected). LH, left hemisphere; RH, right hemisphere.

**FIGURE 4:**
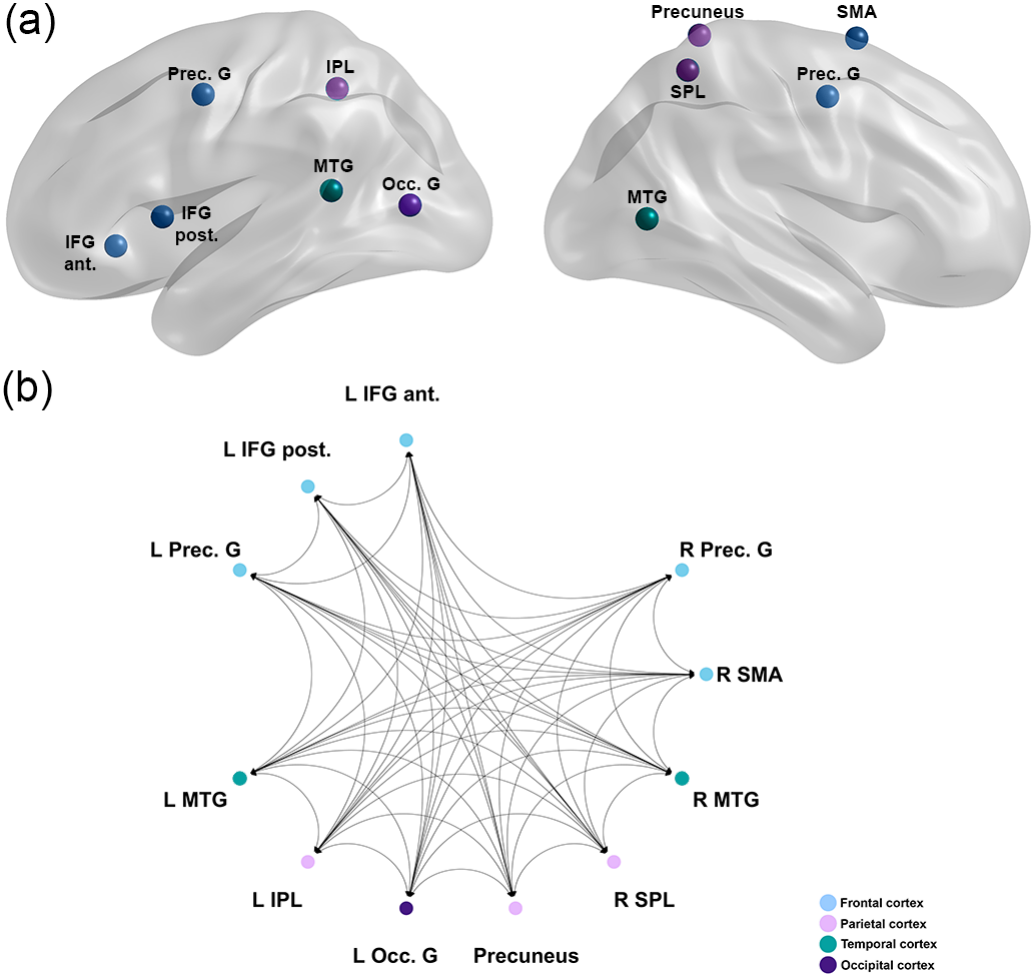
(a) Nodes are used in the spectral dynamic causal modeling (spDCM) analyses. The time series from 11 regions of interest (ROIs) were used to invert the spDCMs with fully connected intrinsic architecture. (b) The initial model assumed a fully connected intrinsic architecture, comprising of the connectivity among each node (i.e., 11^2^ = 121 parameter model). IFG ant., anterior inferior frontal gyrus; IFG post., posterior inferior frontal gyrus; SMA, supplementary motor area; Prec. G, precentral gyrus; IPL, inferior parietal lobe; MTG, middle temporal gyrus; SPL, superior parietal lobe; Occ. G, occipital gyrus; L, left; R, right.

Having validated that the parametric intensity analysis generally returned meaningful results, in a second step, we aimed to determine brain regions that were modulated selectively by stimulus intensity during the down‐regulation of emotions, which was our crucial condition to identify regions‐of‐interest (ROIs) for the following analyses. For this, we exclusively masked the parametric whole‐brain contrasts of stimulus intensity during the *Decrease* condition by the *Look* condition. This contrast yielded increased activity in 11 regions: left anterior and posterior inferior frontal gyrus (L IFG ant. and L IFG post.), bilateral precentral gyri (L Prec. G and R Prec. G), the right supplementary motor area (R SMA), bilateral middle temporal gyri (L MTG and R MTG), the right superior parietal lobe (R SPL), the left inferior parietal lobe (L IPL), the precuneus, and the left occipital gyrus (L Occ. G) (Figure 3). Of note, given that the L IFG exhibits non‐uniform characteristics anatomically, cytoarchitectonically, and functionally (Amunts et al., 1999; Bulut, 2022; Clos et al., 2013; Hagoort & Indefrey, 2014; Rodd et al., 2015; Zaccarella et al., 2017), ROIs in the anterior and posterior part of L IFG have been selected separately for the subsequent spDCM analysis. All these regions were used as ROIs for the subsequent spDCM analysis of the resting‐state fMRI data and are illustrated in Figure 4a. The coordinates are reported in detail in Table 1.

#### 3.2.2 Effective connectivity related to reappraisal success

**FIGURE 5:**
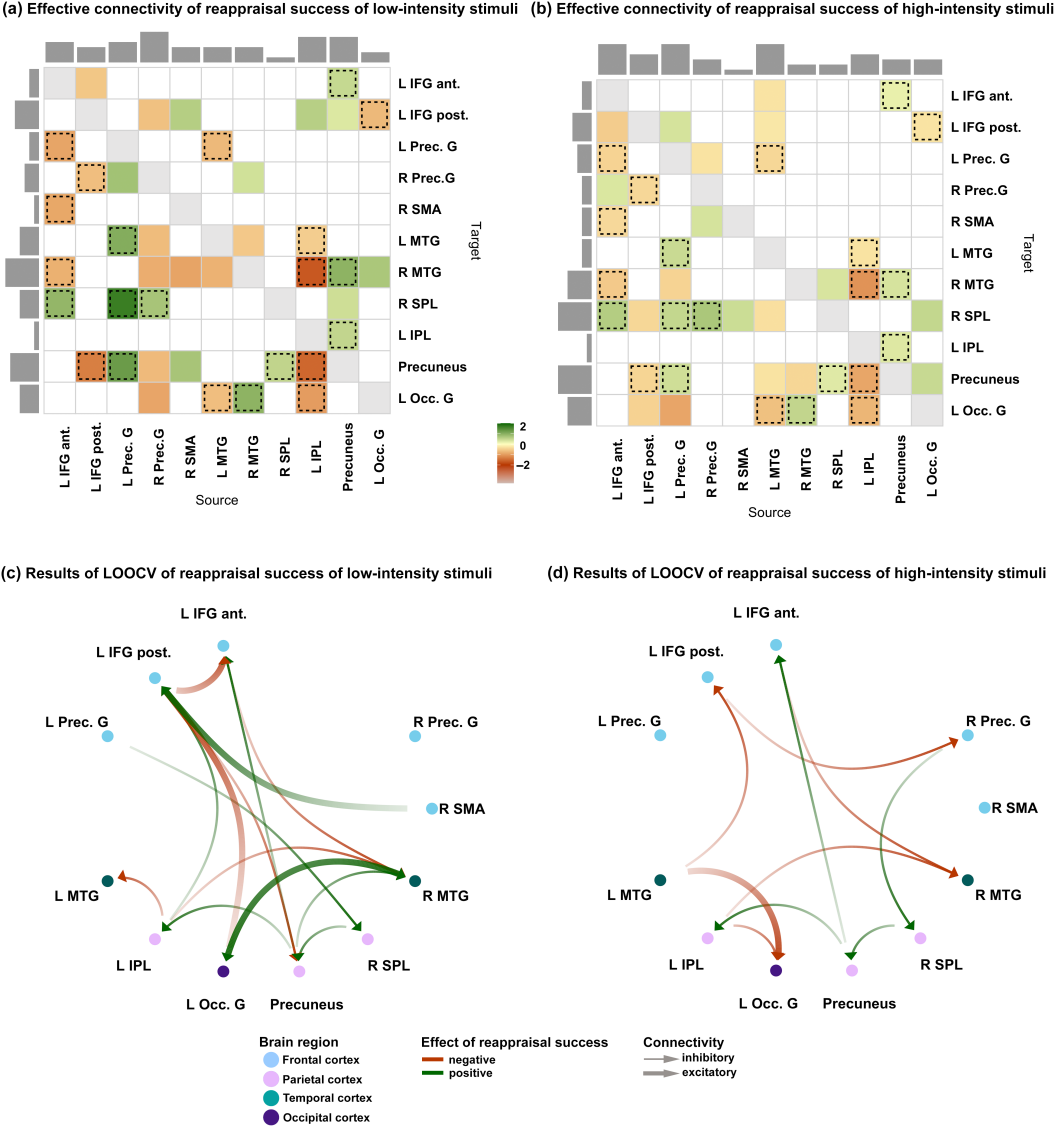
(a,b) Spectral dynamic causal modeling (spDCM) results in effective connectivity of reappraisal success in response to low‐intensity stimuli (a) and high‐intensity stimuli (b). Green/red colors indicate positive/negative connectivity for reappraisal success. Effect sizes that survived a 95% posterior confidence criterion or more are shown in color. The matrix can be interpreted as effective connectivity from column (source) to row (target). Dashed squares mark the overlap of connectivities modulated by reappraisal success in response to low‐ (a) and high‐intensity stimuli (b). (c,d) Leave‐one‐out cross‐validation (LOOCV) results for reappraisal success in response to low‐intensity (c) and high‐intensity (d) stimuli. Only significant effect sizes that were large enough to predict reappraisal success with an out‐of‐sample estimate are illustrated (*p* < .05). Green/red arrows indicate a positive/negative relationship with reappraisal success. The thickness of the arrows indicates excitatory/inhibitory effective connectivity. ROIs are color‐coded for various brain regions. LH, left hemisphere; RH, right hemisphere; IFG ant., anterior inferior frontal gyrus; IFG post., posterior inferior frontal gyrus; SMA, supplementary motor area; PreCG, precentral gyrus; IPL, inferior parietal lobe; MTG, middle temporal gyrus; SPL, superior parietal lobe; Occ. G., occipital gyrus.

The spDCM analyses of the resting state data focused on the modulation of changes in effective connectivity by reappraisal success for high‐ and low‐intensity stimuli, respectively. Therefore, two Parametric Empirical Bayes analyses were conducted using reappraisal success of either high‐intensity or low‐intensity stimuli as predictor for the effective connectivity of the defined neural network of interest. In total, 39 connections between the 11 ROIs were modulated by reappraisal success in response to low‐intensity stimuli, while 40 connections were modulated in response to high‐intensity stimuli. Slightly more connections were inhibitory than excitatory. The results of the relationship between reappraisal success for high‐ and low‐intensity stimuli and respective effective connectivity between the ROIs are shown in Figure 5a,b. Positive values (indicated in green) represent a positive relationship between the effective connectivity of two ROIs and reappraisal success, while negative values (indicated in red) represent a negative relationship. The direction of effective connectivity (i.e., excitatory or inhibitory) between two regions is reported in detail in Tables 3 and 4.

**TABLE 3:** Direction of effective connectivity between two regions using reappraisal success in response to high‐intensity stimuli as covariate.

Direction of connectivity			Interaction with covariate	Effect size in Hz
Source		Target		
Inhibition				
L IFG ant.	→	R MTG	−	−0.54^a^
L IFG post.	→	Precuneus	−	−0.42^a^
L IFG post.	→	L occipital gyrus	−	−0.45^a^
L IFG post.	→	R precentral gyrus	−	−0.39^a^
L IPL	→	R MTG	−	−1.10^a^
L IPL	→	L MTG	−	−0.29^a^
L IPL	→	L occipital gyrus	−	−0.75^a^
L MTG	→	L IFG post.	−	−0.24
L MTG	→	R SPL	−	−0.32
L MTG	→	L precentral gyrus	−	−0.40^a^
L MTG	→	Precuneus	−	−0.29
R MTG	→	Precuneus	−	−0.41
L precentral gyrus	→	L occipital gyrus	−	−0.91
L precentral gyrus	→	R MTG	−	−0.44
L IFG ant.	→	R SPL	+	0.63^a^
L IFG ant.	→	R precentral gyrus	+	0.31
L precentral gyrus	→	R SPL	+	0.51^a^
L precentral gyrus	→	Precuneus	+	0.41^a^
R precentral gyrus	→	R SPL	+	0.71^a^
Precuneus	→	R MTG	+	0.34^a^
Precuneus	→	L IFG ant.	+	0.19^a^
Precuneus	→	L IPL	+	0.28^a^
R SPL	→	Precuneus	+	0.26^a^
Excitation				
L IFG ant.	→	L precentral gyrus	−	−0.44^a^
L IFG ant.	→	R SMA	−	−0.38^a^
L IFG ant.	→	L IFG post.	−	−0.52
L IFG post.	→	R SPL	−	−0.40
L IPL	→	Precuneus	−	−0.90^a^
L MTG	→	L occipital gyrus	−	−0.63^a^
L MTG	→	L IFG ant.	−	−0.27
L occipital gyrus	→	L IFG post.	−	−0.27^a^
R precentral gyrus	→	L precentral gyrus	−	−0.28
R MTG	→	L occipital gyrus	+	0.52^a^
L occipital gyrus	→	R SPL	+	0.51
L occipital gyrus	→	Precuneus	+	0.47
L precentral gyrus	→	L IFG post.	+	0.36
L precentral gyrus	→	L MTG	+	0.46^a^
R precentral gyrus	→	R SMA	+	0.35
R SMA	→	R SPL	+	0.43
R SPL	→	R MTG	+	0.33

^a^ Overlap with low‐intensity stimuli.

**TABLE 4:** Direction of effective connectivity between two regions using reappraisal success in response to low‐intensity stimuli as covariate.

Direction of connectivity			Interaction with covariate	Effect size in Hz
Source		Target		
Inhibition				
L IFG ant.	→	R MTG	−	−0.80^a^
L IFG post.	→	Precuneus	−	−1.32^a^
L IFG post.	→	R precentral gyrus	−	−0.65^a^
L IPL	→	R MTG	−	−1.69^a^
L IPL	→	L MTG	−	−0.50^a^
L IPL	→	L occipital gyrus	−	−1.00^a^
L MTG	→	R MTG	−	−0.78
L MTG	→	L precentral gyrus	−	−0.71^a^
R MTG	→	L MTG	−	−0.56
R precentral gyrus	→	L occipital gyrus	−	−0.91
R precentral gyrus	→	L MTG	−	−0.69
R precentral gyrus	→	Precuneus	−	−0.70
R precentral gyrus	→	R MTG	−	−0.82
R precentral gyrus	→	L IFG post.	−	−0.63
L IFG ant.	→	R SPL	+	0.92^a^
L IPL	→	L IFG post.	+	0.60
R MTG	→	R precentral gyrus	+	0.38
L precentral gyrus	→	R SPL	+	1.68^a^
L precentral gyrus	→	Precuneus	+	1.27^a^
R precentral gyrus	→	R SPL	+	0.74^a^
Precuneus	→	R SPL	+	0.41
Precuneus	→	R MTG	+	0.97^a^
Precuneus	→	L IFG ant.	+	0.48^a^
Precuneus	→	L IPL	+	0.52^a^
Precuneus	→	L IFG post.	+	0.29
R SPL	→	Precuneus	+	0.53^a^
Excitation				
L IFG ant.	→	L precentral gyrus	−	−0.91^a^
L IFG ant.	→	R SMA	−	−0.89^a^
L IFG post.	→	L IFG ant.	−	−0.57
L IPL	→	Precuneus	−	−1.55^a^
L MTG	→	L occipital gyrus	−	−0.65^a^
L occipital gyrus	→	L IFG post.	−	−0.70^a^
R SMA	→	R MTG	−	−0.93
R MTG	→	L occipital gyrus	+	0.97^a^
L occipital gyrus	→	R MTG	+	0.71
L precentral gyrus	→	R precentral gyrus	+	0.78
L precentral gyrus	→	L MTG	+	1.05^a^
R SMA	→	L IFG post.	+	0.62
R SMA	→	Precuneus	+	0.74

^a^ Overlap with high‐intensity stimuli.

#### 3.2.3 Commonalities in effective connectivity between reappraisal of high‐ and low‐intensity stimuli (stimulus intensity independent effects)

Comparing the intrinsic network architecture in relation to reappraisal success between low‐ and high‐intensity stimuli revealed that 56% of all connections were modulated by reappraisal success in both conditions. This is illustrated by the rectangles in Figure 5a,b and indicated by asterisk in Tables 3 and 4. This means that more than half of the connections demonstrated the exact same relationship with reappraisal success *independent* of the stimulus intensity. Thus, this modulation could be interpreted as a general modulation of the intrinsic network architecture by reappraisal success.

Overall, more top‐down connections were modulated by reappraisal success independent of the stimulus intensity (from frontal to temporal, frontal to parietal, and frontal to occipital cortex). In addition, connections from parietal to temporal cortex and from temporal to occipital cortex were related to reappraisal success. The connections from occipital cortex to frontal and temporal regions were also modulated by reappraisal success. Regarding the direction of the connectivity, slightly more connections were inhibitory than excitatory in nature (15 inhibitory and 7 excitatory). However, among those, the relationship with reappraisal success was nearly equally distributed between positive and negative associations.

#### 3.2.4 Differences in effective connectivity between reappraisal of high‐ and low‐intensity stimuli (stimulus intensity dependent effects)

The differences between the two heat maps in Figure 5a,b reveal the intensity‐specific modulation of the intrinsic network dynamics between ROIs by reappraisal success. Overall, 17 connections were distinctly modulated by reappraisal success only in response to low‐intensity stimuli, of which 11 were inhibitory, and six were excitatory. Again, no clear pattern for the type of association (positive or negative) with reappraisal success was found. In response to high‐intensity stimuli, 18 connections were distinctively modulated by reappraisal success, of which 8 connections were inhibitory and 10 were excitatory. Most were negatively related to reappraisal success within the inhibitory connections, while the excitatory connections were equally positively or negatively related to reappraisal success. Specific differences in effective connectivity patterns in relation to stimulus intensity were found for connections from frontal to temporal cortex, demonstrating a negative association with reappraisal success in response to low‐intensity stimuli. Strikingly, the reverse pattern was found for high‐intensity stimuli, that is, connections from temporal to frontal cortex were negatively related to reappraisal success.

Several hubs could be determined by analyzing the input and output ratio of the ROIs (≥5 connections). In the low‐intensity network architecture, the right precentral gyrus, the left IPL, and the precuneus represented regions with the highest outputs, while for the high‐intensity network the left anterior IFG, the left precentral gyrus, and the left MTG provided the most output. The most targeted regions within the low‐intensity network were the left posterior IFG, the right MTG, and the precuneus, while for the high‐intensity network the right MTG, the right SPL, the left occipital gyrus, and the precuneus were most often targeted. These hubs might be critical for regulating the flow and integration of information within networks.

#### 3.2.5 Prediction of reappraisal success based on intrinsic network connectivity

Leave‐one‐out cross‐validation (LOOCV) was conducted to explore which specific connections within the network could significantly predict reappraisal success across participants depending on stimulus intensity. The LOOCV revealed that effect sizes were large enough to predict reappraisal success with an out‐of‐sample estimate for 15 connections for low‐intensity (Figure 5c and Table 5) and 10 connections for high‐intensity stimuli, respectively (Figure 5d and Table 5). Five of these predictive connections were independent of stimulus intensity, meaning that these were predictive in both the low‐ and high‐intensity networks. Overall, higher predictability of reappraisal success was found for more connections for low‐intensity stimuli (10 connections) compared with high‐intensity stimuli (5 connections). Notably, within the low‐intensity network, the inhibitory connections from the precuneus to the frontal and parietal cortex and from the parietal cortex to the precuneus were the most predictive of reappraisal success (positive association).

**TABLE 5:** Results of LOOCV analyses.

Source		Target	*r*‐value	*p*‐value
Reappraisal success in response to high‐intensity stimuli				
L IFG ant.	→	R MTG	.32	.05^a^
L IFG post.	→	R precentral gyrus	.33	.04
L IPL	→	R MTG	.40	.02^a^
L IPL	→	L occipital gyrus	.33	.04
L MTG	→	L IFG post.	.34	.04
L MTG	→	L occipital gyrus	.35	.03
R precentral gyrus	→	R SPL	.34	.04
Precuneus	→	L IFG ant.	.36	.03^a^
Precuneus	→	L IPL	.42	.01^a^
R SPL	→	Precuneus	.32	.05^a^
Reappraisal success in response to low‐intensity stimuli				
L IFG ant.	→	R MTG	.36	.03^a^
L IFG post.	→	Precuneus	.37	.03
L IFG post.	→	L IFG ant.	.33	.04
L IPL	→	R MTG	.34	.04^a^
L IPL	→	L MTG	.34	.04
L IPL	→	L IFG post.	.33	.04
R MTG	→	L occipital gyrus	.33	.04
L occipital gyrus	→	L IFG post.	.37	.03
L occipital gyrus	→	R MTG	.34	.04
L precentral gyrus	→	R SPL	.33	.04
Precuneus	→	R MTG	.50	<.001
Precuneus	→	L IFG ant.	.56	<.001^a^
Precuneus	→	L IPL	.50	<.001^a^
R SMA	→	L IFG post.	.32	.05
R SPL	→	Precuneus	.37	.03^a^

^a^ Overlap between high‐intensity and low‐intensity stimuli.

## 4 DISCUSSION

In this study, we used spectral dynamic causal modeling to characterize the temporal dynamics of brain connectivity at rest in a network of regions, which were shown to be parametrically modulated by stimulus intensity in a separate emotion regulation task. Within this intensity‐modulated brain network, we predicted reappraisal success from the intrinsic network dynamics at rest, separately for high‐ and low‐intensity stimuli. Specifically, we found that overall changes in connectivity strength from frontal regions to the parietal, temporal, and occipital cortex were related to reappraisal success independent of the stimulus intensity. We observed differences in effective connectivity in relation to low‐intensity stimuli specifically for motor regions connecting to frontal and temporal regions. Reappraisal success in response to high‐intensity stimuli was predicted by additional connections within the vlPFC and from temporal to frontal regions. These findings provide strong evidence that the network dynamics at rest are not random, but provide meaningful insights into future task performance in emotion regulation. Our findings provide a first detailed picture of the specific directionality of predictive connectivity patterns at rest. We further show that different individual connectivity dynamics might be suited to regulating high‐intensity or low‐intensity stimuli, demonstrating a crucial interaction between individuals' network dynamics and their ability to adequately react to different contextual emotional challenges.

### 4.1 Brain activation related to parametric modulation of stimulus intensity and emotional state

First, we will address the implications of the task‐related fMRI results, which are interesting in their own right. This is the first study to reveal how trial‐by‐trial variability in stimulus intensity influences neural responses during reappraisal. One previous study failed to report significant effects on a whole‐brain level using parametric analysis but provided first insights into how stimulus intensity affects brain activity, mainly in the lateral prefrontal cortex (Silvers et al., 2015). Although we used a highly similar task design, our study differed from the study of Silvers et al. (2015) as two crucial issues strongly increased the sensitivity of our analyses. First, we used ultra‐high field strength (7 T compared with 3 T), and second, we implemented multiple scanning sessions (3 sessions compared with 1), thereby significantly increasing the number of total experimental trials (*n* = 240 trials compared with *n* = 108 trials).

From a behavioral perspective, one would expect that emotional state ratings and stimulus intensity are directly negatively related (Rammensee et al., 2023), that is, experiencing more negative emotions is associated with increased stimulus intensity. This was confirmed by our correlation analysis. On a neuronal level, higher stimulus intensity was related to increased brain activity in emotion generation and regulation regions (Morawetz et al., 2020; Morawetz, Bode, Baudewig, & Heekeren, 2017), while feeling less negative after regulation was related to decreased activity within the same regions. This finding is in line with top‐down models of emotion regulation, where it has been shown that a decrease in amygdala responses was related to a decrease in emotional state ratings (e.g., Eippert et al., 2007; Ochsner et al., 2009; Paschke et al., 2016; Winecoff et al., 2011). We extend these findings by demonstrating that these effects are not restricted to the amygdala, but that a wide range of cortical and subcortical regions were modulated in the same way by the emotional state (e.g., McRae et al., 2010; Silvers et al., 2016a, 2016b; Wager et al., 2008) and more significantly, also by stimulus intensity (including IFG, MTG, thalamus, SMG, IPL, cingulate cortex, precentral gyrus). These regions have been discussed to be involved in language processing to actively reinterpret the meaning of the emotional stimulus and the selection of goal‐appropriate reappraisals as well as emotional reactivity (Badre & Wagner, 2007; De La Vega et al., 2018; Morawetz et al., 2020).

When analyzing reappraisal‐specific regions by controlling for the control condition in the parametric contrast of emotion regulation, the trial‐by‐trial variability in stimulus intensity specifically modulated activity in frontal, temporal, and parietal regions, SMA and precuneus. As the activity in these reappraisal‐related regions specifically increases with stimulus intensity, this might indicate an increase in cognitive demand and stronger recruitment of the whole network to effectively implement reappraisal (Morawetz et al., 2020; Morawetz, Bode, Baudewig, & Heekeren, 2017).

### 4.2 Commonalities in effective connectivity between reappraisal of high‐ and low‐intensity stimuli

The present findings are the first to demonstrate the underlying causal network dynamics associated with reappraisal success in response to both high‐ and low‐intensity stimuli. Independent of the stimulus intensity, several regions of the determined network demonstrated less connectivity with increasing reappraisal success. Nearly all frontal connections to the rest of the brain were part of this core network. For example, inhibitory connectivity at rest from the prefrontal cortex to temporal and motor cortex regions was negatively related to reappraisal success. The same association was found for inhibitory connections from the parietal cortex to temporal regions. In addition, an increase in excitatory connectivity from the prefrontal cortex to motor regions was related to less reappraisal success. In contrast, several regions also demonstrated the opposite effect, meaning that greater effective connectivity was related to increased reappraisal success. These connections included connectivity from precentral gyrus to parietal and temporal regions. A positive association to reappraisal success was also found for the inhibitory connectivity from the precuneus to the parietal, temporal, and frontal regions.

These findings suggest that the overall decrease in inhibition mediated by the vlPFC, motor and parietal cortex, and precuneus is a prerequisite for effective reappraisal. Through the widespread connectivity, the lateral PFC receives input from the motor cortex, temporal, and parietal cortex (Petrides, 2005; Petrides & Pandya, 1999, 2002a, 2002b). When actively regulating emotions, these regions would provide the lateral PFC with highly processed information relevant for the high‐level appraisals related to emotion regulation (Dixon et al., 2017). This includes information about current goals, contextual information, actions, and expected outcomes. In addition, the precuneus which has been implicated in episodic memory retrieval and self‐processing operations, supports effective reappraisal by serving first‐person perspective‐taking and generating an experience of agency (Cavanna & Trimble, 2006). This interaction between top‐down and bottom‐up regions is consistent with the theoretical models of emotion that suggest continuous bidirectional interactions to enable the appraisal process (Barrett & Satpute, 2013; Dixon et al., 2017; Gross, 2015; Lewis, 2005; Scherer, 2001; Smith & Lane, 2015). Thus, the dampened inhibitory state at rest might represent a preparatory state needed to ensure effective emotion regulation (Chauvin et al., 2019) reflected in bidirectional feedback loops along the processing hierarchy from stimulus input to action output.

Previous work proposed that several processes governed by different neural networks are likely invoked in successful emotion regulation, including top‐down modulation of attention, self‐conscious recall of an intended action, and sensory processing of the attended cue (Seo et al., 2014). Importantly, in our study the effective connectivity was not measured during the task, so it remains speculative how strongly our results reflect preparedness for these specific cognitive processes. We also did not assess whether the same network configuration predicted regulation success for other strategies than reappraisal, that is, whether they generalize to emotion regulation more broadly. It is entirely possible that an individual's effective connectivity at rest that predicts poor reappraisal success might also predict better success for using, for example, distraction as a strategy. However, at least for reappraisal, the present findings support the notion that successful emotion regulation indeed relies on specific dynamic network configurations between top‐down and bottom‐up emotion regulatory and generating networks at rest. This, in turn, suggests that relatively stable individual differences exist in the tuning of this network that generalize across contextual circumstances (i.e., different emotional intensities) that are directly related to how well individuals will respond to an emotionally challenging situation.

### 4.3 Differences in effective connectivity between reappraisal of high‐ and low‐intensity stimuli

Interestingly, in addition to a common network predicting success for reappraisal of both high‐ and low‐intensity stimuli, there were also important differences in the network dynamics for these two stimulus classes. An increase in reappraisal success in response to low‐intensity stimuli was predicted by decreased inhibitory connectivity from regions in the right precentral gyrus to most of the other included regions. In addition, greater inhibitory connectivity from the precuneus to the parietal and frontal cortex regions was related to increased reappraisal success. The precuneus has been previously related to self‐reflection, self‐conscious mental processes, and attentive control (Cavanna & Trimble, 2006; Den Ouden et al., 2005; Takahashi et al., 2015), suggesting a state of enhanced conscious, attention‐driven processing of internal goals (Ferri et al., 2016). One speculative explanation for why this connectivity was particularly relevant for low‐intensity stimuli might be that low‐intensity stimuli afforded participants with more cognitive resources to reflect on the self‐relevance of these stimuli. Given the instructions for reappraisal, this specific connectivity pattern at rest might reflect a neural preparedness to distance oneself from stimuli by perceiving them as “not related to oneself.” This might have been easier (and more consistent) for stimuli that did not elicit an overwhelming negative emotional response (i.e., are of lower intensity).

In contrast, increased reappraisal success in response to high‐intensity stimuli was related to decreased inhibitory connectivity from the temporal cortex to the frontal, parietal, and occipital regions. In particular, the right precentral gyrus has been associated with the modulation of sustained attention, and self‐related awareness (Cabeza & Nyberg, 2000; Théoret et al., 2004), suggesting that this region is engaged in modulating affect‐driven attention. The temporal cortex regions, whose connectivity was important for high‐intensity stimuli, have been implicated in modulating anticipatory attention in cue‐related processing (Corbetta & Shulman, 2002; Serences & Yantis, 2006). A speculative explanation for this pattern might be that less connectivity between the temporal regions and other areas, in particular prefrontal control regions, allowed participants to reduce the impact of bottom‐up attention‐capturing effects that high‐intensity stimuli naturally elicit. In other words, cue‐related (i.e., stimulus‐related) effects of negative high‐intensity stimuli might be easier to deal with during reappraisal if this effective connectivity is already weaker at rest.

Notably, an interesting difference in effective connectivity was also found within the prefrontal cortex. For low‐intensity stimuli, less excitatory connectivity at rest from the posterior to the anterior part of the IFG predicted increased reappraisal success. However, the reverse pattern was observed for the high‐intensity condition, where less excitatory connectivity from the anterior to the posterior part of the IFG was associated with increased reappraisal success. These results indicate that projections within different parts of the vlPFC might be specifically modulated by emotional intensity. Previous studies on language demonstrated functional segregation within the left IFG, with semantics, syntax, phonological processing and verbal working memory represented in different subregions of the IFG (Bookheimer, 2002; Friederici, 2009; Hagoort, 2005; Ishkhanyan et al., 2020; Tyler et al., 2011). The anterior part of the left IFG has been associated with semantic processing both during language comprehension and production. In contrast, the posterior part of the IFG has been suggested to be engaged in syntactic aspects of language comprehension and production as well as picture naming (Krieger‐Redwood & Jefferies, 2014). Given the fact that IFG in particular has been associated with attaching verbal labels to emotions (Lieberman et al., 2007) and together with MTG and IPL has been linked to self‐directed inner speech (Jones & Fernyhough, 2007; Luria, 1962; Shergill et al., 2002; Vygotsky, 1962), our findings could be interpreted in the light of the usage‐based linguistic theory (Boye & Bastiaanse, 2012). This theory posits that the distinction between grammatical (linked to the posterior IFG) and lexical (linked to the anterior IFG) information is based on a semantic distinction about information prominence: Lexical information highlights the main point of a statement, that is, foreground information, which is more detailed and specific and conveys the main point and is represented in the anterior IFG. Grammatical information is secondary information, that is, background information provided by the posterior IFG. The shift in connectivity between the anterior and posterior part of the IFG might reflect the relevance of the processing of foreground and background information for the reappraisal depending on the context: High‐intensity stimuli are more complex to reappraise, and thus, it is important to have a detailed reinterpretation of the meaning of the stimulus, making the foreground information integrated by the anterior IFG more relevant. Low‐intensity stimuli are easier to reappraise, and even general statements might be good enough to change their emotional meaning, moving background information more to the foreground. We note that all these ideas, of course, now have to be tested in more targeted functional studies. However, our findings now provide a foundation for these hypotheses. In summary, our findings point towards functional segregation of the vlPFC concerning the contextual demands during effective emotion regulation.

Overall, reappraisal success for low‐intensity stimuli was better predicted from resting‐state effective connectivity than for high‐intensity stimuli. This could be related to the fact that low‐intensity stimuli were easier to regulate and rely on the preparedness reflected in more stereotyped connectivity patterns. Previous research indicated that highly intense emotions might be associated with a greater need for regulation (Feldman Barrett et al., 2010), and that when participants can choose, they less frequently use reappraisal in high‐intensity situations (Dixon‐Gordon et al., 2015; Suri et al., 2014; Wilms et al., 2020). Therefore, for high‐intensity stimuli, the general network dynamics might be relatively less important compared with low‐intensity stimuli, while other factors during task performance might play a stronger role. These could include task‐related connectivity and the flexible recruitment of additional cognitive resources. Some support for this idea comes from our whole‐brain parametric analysis, which demonstrated an increase in brain activity during the implementation of reappraisal with an increase in negative emotional state. This, in turn, might be related to the formation of a mini‐network, so‐called process‐specific alliances (PSA), that rapidly assemble to mediate a cognitive process in response to task demands (Cabeza et al., 2018). It has been suggested that emotion regulation is based on a PSA, with the vlPFC as a key player. Assuming that emotionally challenging situations demand flexible and temporary recruitment of a frontal‐based PSA, this might be reflected in increased brain activity during active regulation and explain the reduced importance of specific effective connectivity patterns at rest (Greene et al., 2018; Zhao et al., 2023). Thus, our findings align well with the multiple large‐scale network account in affective science that assumes that emotional experience is based on dynamic and interactive brain networks (Barrett & Satpute, 2013; Morawetz et al., 2020; Morawetz, Alexandrowicz, & Heekeren, 2017; Pessoa, 2018; Riedel et al., 2018; Smith & Lane, 2015). In addition, our findings further support the model of regulatory selection (Sheppes, 2020) and the consideration of contextual factors in the regulation process (Aldao, 2013; Aldao & Nolen‐Hoeksema, 2012; Dore et al., 2016) by demonstrating a clear impact of stimulus‐intensity on underlying resting‐state networks dynamics. Together, our results suggest that resting effective connectivity in reappraisal‐related networks predicts subsequent reappraisal success, and that under challenging conditions, these regions are dynamically recruited to implement reappraisal effectively.

## 5 LIMITATIONS

We note that there are several limitations to consider when interpreting our findings. First, we did not investigate task‐based connectivity but focused on resting‐state connectivity only. This, however, is a widely overlooked factor that might be diagnostic for the neural preparedness for successful emotion regulation ability. Since emotion regulation ability represents a transdiagnostic feature in psychopathology (Aldao et al., 2016; Cludius et al., 2020; Kring & Sloan, 2010; Sloan et al., 2017), it is important to determine biological markers to predict emotion regulation ability (Horwitz & Rowe, 2011). Our study is a first step towards this goal, providing evidence that resting‐state connectivity has the potential to serve as such a predictor. However, future research should also aim to investigate the relationship between effective connectivity at rest and task‐related effective connectivity, to better understand how the neural preparedness for emotion regulation might be translated to network dynamics during task performance (Cole et al., 2021).

Our findings are further limited by the fact that we only examined reappraisal. It remains unknown whether our results can be generalized to other emotion regulation strategies such as distraction or suppression (Dörfel et al., 2014). Some of the predictive connectivity patterns discussed above might indeed be related to how easy or hard it was to implement reappraisal for specific stimulus classes (Scheffel et al., 2021), for example, whether participants could use inner speech, or process the self‐relevance of the stimuli. Such research would also allow for understanding whether specific network dynamics at rest could predispose people to use suboptimal strategies.

The sequencing of the emotion regulation task before the resting‐state data collection could potentially introduce biases or alterations in resting‐state brain activity and connectivity. The interaction between resting‐state activity/connectivity and task‐based activity can progress in either of two directions (Lor et al., 2023; Northoff et al., 2010). Emotion regulation, being a dynamic process, might induce lingering effects that could influence the subsequent resting‐state measurements by altering this basal level of activity and connectivity in some way. Conversely, resting‐state connectivity could interact with task‐based activity, thus impacting the reappraisal success. Several studies provide evidence that task‐based activity could modulate resting‐state activity (Cecchetto et al., 2019; Han et al., 2008; Lewis et al., 2009; Newton et al., 2007; Sarabi et al., 2018). On the other hand, there is strong evidence that resting‐state connectivity could shape cognitive task activations and predict brain activity during task performance. A high degree of similarity between task‐based and resting‐state functional connectivity has been observed (Cole et al., 2016, 2021; Smith et al., 2009; Tavor et al., 2016). This suggests that the functional network architecture identified using resting‐state functional connectivity might be related to network connectivity during task performance, and could plausibly reflect the routes by which activity flows during cognitive task performance. In future work, implementing a more controlled experimental design, such as introducing a washout period between the task and resting‐state acquisition, or collecting the resting‐state data before the task‐based runs, could help address these questions.

Another limitation is that we only investigated healthy adults, which might limit the generalizability to other populations. Our sample size was also not large; however, it must be noted that we collected three times as much data as regular studies, compensating for this limitation (Desmond & Glover, 2002). Therefore, future studies should include larger and more diverse samples. There are several clinical groups, however, that show emotional dysregulation, such as evident in borderline, personality disorder, substance use disorder, depression, and anxiety disorder (Aldao et al., 2010, 2016; Eftekhari et al., 2009; Gross & Jazaieri, 2014; Taylor & Liberzon, 2007; Werner & Gross, 2010). Importantly, the ability to regulate one's emotion further develops over the lifespan, with adolescence being a crucial period that is marked by changes in the connectivity within and between the prefrontal cortex and the amygdala, with the amygdala also demonstrating a decrease in reactivity (Silvers et al., 2012, 2015, 2016a, 2017). There might be the potential to use a resting state effective connectivity approach as a tool for early discovery of developmental delays (McGrath et al., 2023), which then could be potentially targeted by emotion regulation training (Denny, 2018; Denny et al., 2015; Denny & Ochsner, 2014). Therefore, future studies should include larger and more diverse samples.

## 6 CONCLUSIONS

We have shown that resting‐state effective connectivity can serve as a predictor for successful reappraisal. We found both common and specific network dynamics predicting reappraisal success for high‐ and low‐intensity stimuli. These appear to reflect specific preparatory states, enabling individuals to respond effectively to emotional stimuli in the future. Our findings can be interpreted as a neural substrate contributing to more vigilant, attentive, self‐aware, and goal‐directed cognitive states that allow for focusing on the regulation of emotions, in particular when using reappraisal to dampen the emotional impact of external stimuli. Our study also provides the first experimental evidence for large‐scale brain dynamics at rest and their link to reappraisal success, and that these differ for emotional stimuli of different intensities. These results may be significant for future research investigating psychopathological alterations in brain connectivity related to affective disorders (Aldao et al., 2016; Cludius et al., 2020; Fernandez et al., 2016; Kring & Sloan, 2010; Sloan et al., 2017).

## CONFLICT OF INTEREST STATEMENT

The authors declare that the research was conducted in the absence of any commercial or financial relationships that could be construed as a potential conflict of interest.

## Supporting information


**Data S1.** Supporting Information.

## Data Availability

The data that support the findings of this study are available from the corresponding author upon reasonable request.
