# Supplementary material for: Resting‐state effective connectivity is systematically linked to reappraisal success of high‐ and low‐intensity negative emotions
Source: Hum Brain Mapp. 2024 Mar 27;45(5):e26667. doi: 10.1002/hbm.26667 (PMC10973777; doi:10.1002/hbm.26667)
Supplement: Supplementary file 1 — Data S1. Supporting Information. [file HBM-45-e26667-s001.docx]

Supplemental Materials

Resting-state effective connectivity is systematically linked to reappraisal success of high- and low-intensity negative emotions

Carmen Morawetz^1^, Stella Berboth^2,^ Stefan Bode^3^

^1^ Department of Psychology, University of Innsbruck, Austria

^2^ Department of Psychiatry and Psychotherapy, Charité - Universitätsmedizin Berlin, Germany

^3^ Melbourne School of Psychological Sciences, The University of Melbourne, Australia

**Contact information**

Carmen Morawetz

Department of Psychology

University of Innsbruck

[carmen.morawetz@uibk.ac.at](mailto:carmen.morawetz@uibk.ac.at)

**Supplemental Figures**


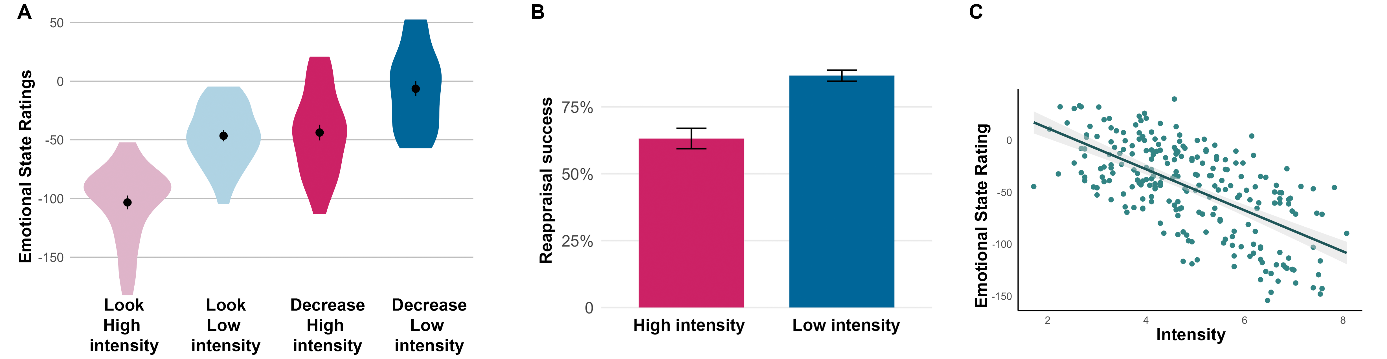


**Supplemental Figure S1. (A)** Emotional state ratings as a function of task condition and stimulus intensity. Participants indicated their emotional state following emotion regulation on a trial-by-trial basis on a scale from -200 (very negative) to +200 (very positive). **(B)** % Reappraisal success as a function of stimulus intensity. **(C)** Pearson correlation between emotional state ratings and stimulus intensity for each stimulus.


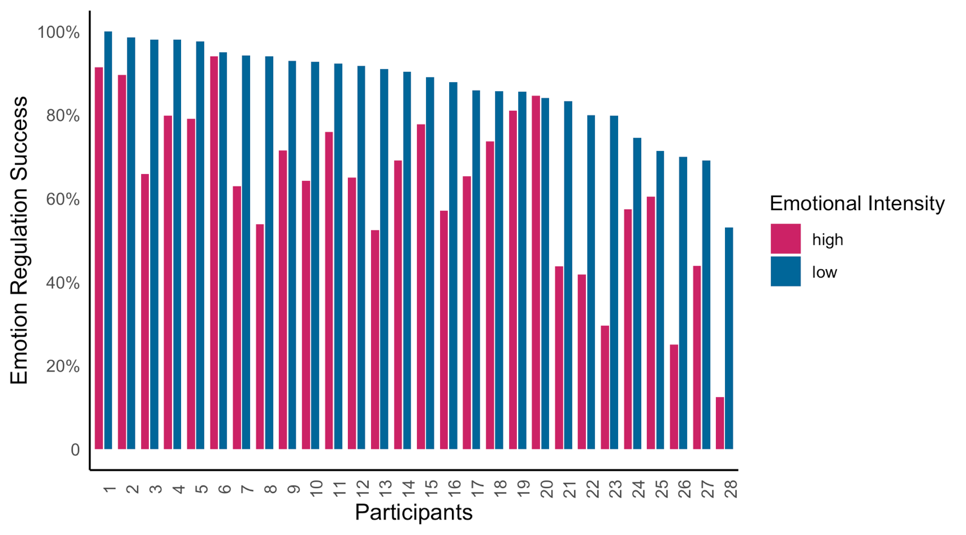


**Supplemental Figure S2.** Original reappraisal success scores in response to high-intensity (purple) and low-intensity (blue) stimuli for each participant.


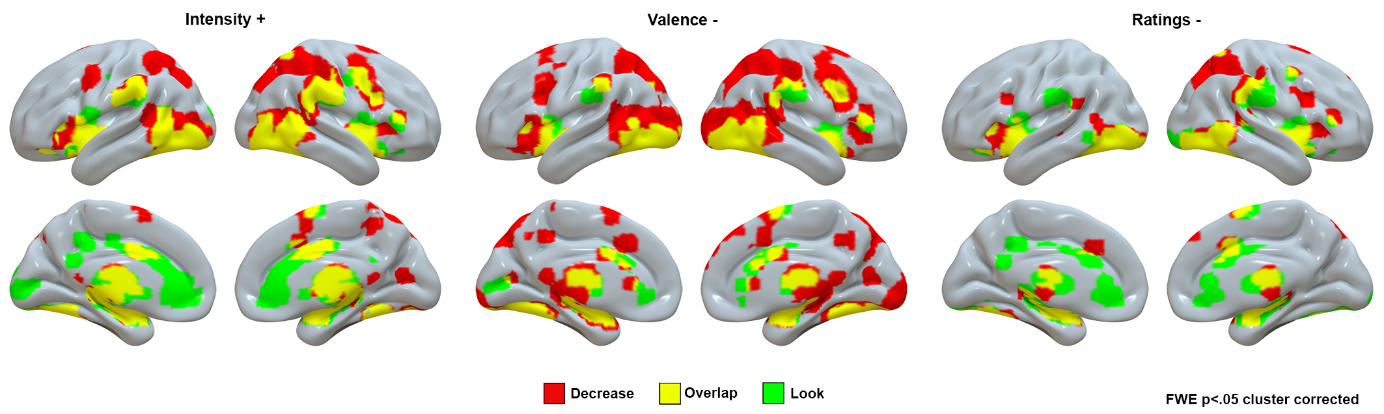


**Supplemental Figure S3.** Results of the whole-brain parametric analyses for stimulus intensity (left), stimulus valence (middle) and emotional state ratings (right). Ratings on stimulus intensity and valence were obtained after the fMRI experiment for each presented image. Emotional state ratings were assessed on a trial-by-trial basis after the emotion regulation during the task-based fMRI. All ratings were used as parametric regressor during the Decrease (indicated in red) and Look (indicated in green) condition during the task-based fMRI session. The overlap in parametrically modulated activity is indicated in yellow. Increased activity within the depicted regions was related to increased stimulus intensity, decreased valence, and decreased emotional state ratings (i.e., more negative feelings). A threshold of FWE p<0.05 cluster corrected was used.


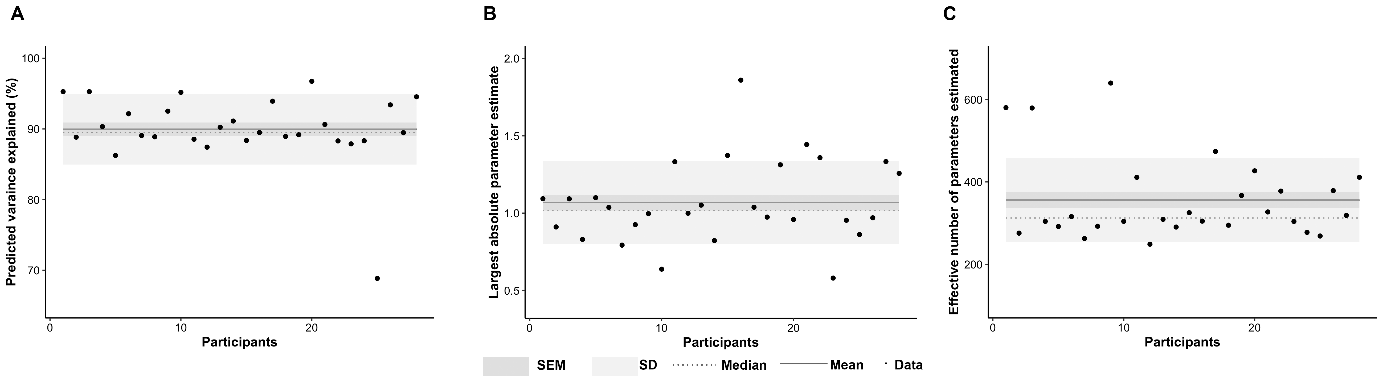


**Supplemental Figure S4.** First level DCM model convergence statistics indicating good model convergence. **(A)** Predicted variance explained for each individual were above the minimum threshold of 10%. **(B)** The largest absolute parameter estimate did not fall below the typical connection strength of 1/8 Hz. **(C)** The effective number of parameters are reported in terms of divergence between the posterior and prior densities over parameters. All post-hoc diagnostics were obtained via *spm_dcm_fMRI_check* (1).


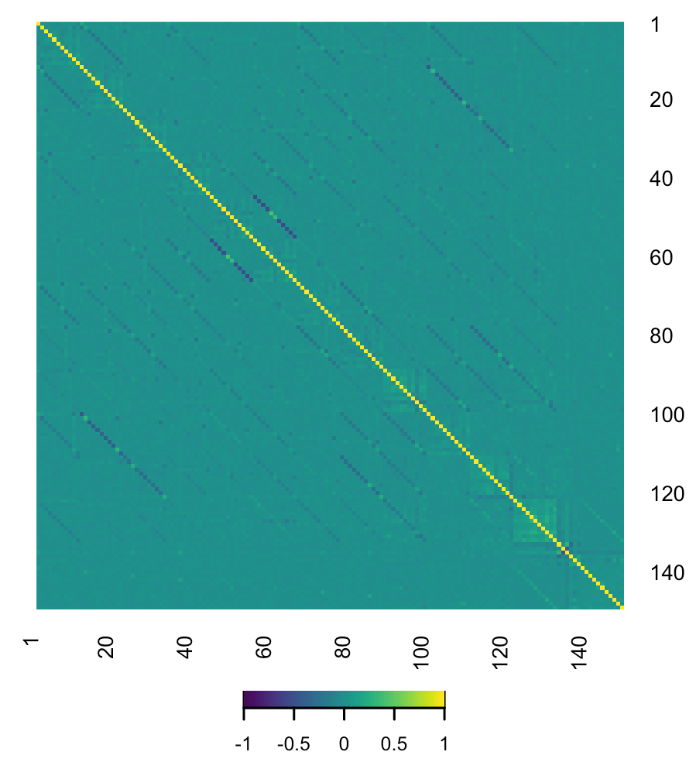


**Supplemental Figure S5.** Posterior correlations among all parameters were low, indicating identifiable parameters. All post-hoc diagnostics were obtained via *spm_dcm_fMRI_check* (1).

**References**

1. Zeidman P, Jafarian A, Seghier ML, Litvak V, Cagnan H, Price CJ, Friston KJ (2019): A guide to group effective connectivity analysis, part 2: Second level analysis with PEB. *Neuroimage* 200: 12–25.

**Supplemental Tables**

| **Table S1. Regions implicated in emotion generation and regulation parametrically modulated by stimulus intensity.** | | | | | | | | |
| --- | --- | --- | --- | --- | --- | --- | --- | --- |
|  |  |  |  |  |  | **Coordinates** | | |
| **Contrast** | **Parametric regressor** | **Region** | **Size** | ***t*-value** | ***p*** | ***x*** | ***y*** | ***z*** |
| *Decrease* | *Intensity +* | Middle Temporal Gyrus | 7328 | 7.22 | <0.001 | -52 | -72 | 4 |
|  |  |  |  | 6.36 |  | -48 | -62 | -16 |
|  |  |  |  | 6.19 |  | -40 | -72 | -14 |
|  |  | Middle Temporal Gyrus | 14098 | 6.77 | <0.001 | 56 | -66 | 4 |
|  |  |  |  | 6.54 |  | 28 | -58 | 64 |
|  |  |  |  | 6.24 |  | 42 | -52 | -10 |
|  |  | Amygdala | 3256 | 6.18 | <0.001 | -21 | -4 | -12 |
|  |  |  |  | 5.23 |  | -33 | -13 | -9 |
|  |  |  |  | 4.83 |  | -32 | 28 | -4 |
|  |  | Precentral Gyrus | 2264 | 5.49 | <0.001 | 46 | 4 | 39 |
|  |  |  |  | 4.53 |  | 33 | -4 | 48 |
|  |  |  |  | 4.31 |  | 45 | 12 | 27 |
|  |  | Amygdala | 3472 | 5.41 | <0.001 | 22 | -2 | -18 |
|  |  |  |  | 5.12 |  | 21 | -6 | -10 |
|  |  |  |  | 4.99 |  | 4 | -28 | -2 |
|  |  | Thalamus | 1647 | 5.29 | <0.001 | 12 | -2 | 6 |
|  |  |  |  | 4.31 |  | -12 | -6 | 12 |
|  |  |  |  | 4.25 |  | 3 | -7 | 9 |
|  |  | Supramarginal Gyrus | 938 | 4.86 | <0.001 | -60 | -37 | 36 |
|  |  |  |  | 3.85 |  | -66 | -22 | 28 |
|  |  |  |  | 3.67 |  | -66 | -30 | 32 |
|  |  | Inferior Parietal Lobe | 1272 | 4.48 | <0.001 | -24 | -48 | 48 |
|  |  |  |  | 4.28 |  | -21 | -60 | 54 |
|  |  |  |  | 3.88 |  | -16 | -66 | 45 |
|  |  | Middle Cingulum | 490 | 4.09 | 0.004 | 2 | 4 | 32 |
|  |  |  |  | 3.58 |  | -2 | 17 | 34 |
|  |  |  |  | 3.56 |  | 6 | 12 | 42 |
|  |  |  |  |  |  |  |  |  |
| *Decrease* | *Intensity -* | no significant clusters |  |  |  |  |  |  |
|  |  |  |  |  |  |  |  |  |
| *Look* | *Intensity +* | Amygdala | 8421 | 7.00 | <0.001 | 21 | -6 | -10 |
|  |  |  |  | 6.76 |  | 27 | -2 | -16 |
|  |  |  |  | 6.75 |  | -20 | -7 | -10 |
|  |  | Thalamus | 1821 | 6.58 | <0.001 | -2 | -7 | 9 |
|  |  |  |  | 4.47 |  | 3 | -19 | 13 |
|  |  |  |  | 4.44 |  | -4 | -7 | -4 |
|  |  | Middle Temporal Gyrus | 3502 | 5.92 | <0.001 | 57 | -58 | 1 |
|  |  |  |  | 5.86 |  | 42 | -49 | -10 |
|  |  |  |  | 5.58 |  | 39 | -54 | -16 |
|  |  | Anterior Cingulum | 3626 | 5.69 | <0.001 | -2 | 34 | -8 |
|  |  |  |  | 4.89 |  | -2 | 36 | 3 |
|  |  |  |  | 4.85 |  | 2 | 17 | 32 |
|  |  | Inferior Temporal Gyrus | 3824 | 5.49 | <0.001 | -45 | -46 | -12 |
|  |  |  |  | 5.33 |  | -36 | -62 | -16 |
|  |  |  |  | 5.28 |  | -36 | -78 | -12 |
|  |  | Supramarginal Gyrus | 1914 | 5.15 | <0.001 | 63 | -37 | 27 |
|  |  |  |  | 5.09 |  | 57 | -24 | 27 |
|  |  |  |  | 4.66 |  | 62 | -30 | 38 |
|  |  | Supramarginal Gyrus | 1036 | 4.90 | <0.001 | -54 | -32 | 27 |
|  |  |  |  | 4.35 |  | -58 | -37 | 36 |
|  |  |  |  | 4.23 |  | -58 | -25 | 27 |
|  |  | Precentral Gyrus | 542 | 4.45 | 0.003 | 54 | 0 | 48 |
|  |  |  |  | 3.90 |  | 46 | 4 | 34 |
|  |  |  |  | 3.79 |  | 39 | -12 | 38 |
|  |  |  |  |  |  |  |  |  |
| *Look* | *Intensity -* | Parahippocampal Gyrus | 595 | 5.84 | 0.002 | 18 | -37 | -11 |
| Coordinates refer to MNI coordinate system. P<0.05 FWE corrected (k=10). + and - indicate a positive or negative association between brain activity and stimulus intensity. | | | | | | | | |

| **Table S2. Regions implicated in emotion generation and regulation parametrically modulated by stimulus valence.** | | | | | | | |  |
| --- | --- | --- | --- | --- | --- | --- | --- | --- |
|  |  |  |  |  |  | **Coordinates** | | |
| **Contrast** | **Parametric regressor** | **Region** | **Size** | ***t*-value** | ***p*** | ***x*** | ***y*** | ***z*** |
| *Decrease* | *Valence +* | no significant clusters |  |  |  |  |  |  |
|  |  |  |  |  |  |  |  |  |
| *Decrease* | *Valence -* | Fusiform Gyrus | 10341 | 8.33 | <0.001 | -39 | -70 | -15 |
|  |  |  |  | 7.58 |  | -34 | -84 | -14 |
|  |  |  |  | 6.88 |  | -38 | -49 | -18 |
|  |  | Fusiform Gyrus | 10765 | 8.03 | <0.001 | 38 | -79 | -14 |
|  |  |  |  | 7.65 |  | 39 | -48 | -20 |
|  |  |  |  | 7.31 |  | 44 | -50 | -12 |
|  |  | Thalamus | 13362 | 6.91 | <0.001 | 22 | -30 | 1 |
|  |  |  |  | 6.37 |  | -27 | -4 | -16 |
|  |  |  |  | 6.27 |  | 24 | -2 | -18 |
|  |  | Inferior Parietal Lobe | 6832 | 6.00 | <0.001 | 32 | -50 | 49 |
|  |  |  |  | 5.78 |  | 30 | -62 | 62 |
|  |  |  |  | 5.70 |  | 26 | -55 | 54 |
|  |  | Precentral Gyrus | 3486 | 5.88 | <0.001 | 45 | 4 | 42 |
|  |  |  |  | 5.50 |  | 38 | 12 | 31 |
|  |  |  |  | 5.18 |  | 48 | 14 | 25 |
|  |  | Superior Parietal Lobe | 2674 | 5.10 | <0.001 | -30 | -55 | 56 |
|  |  |  |  | 4.69 |  | -28 | -49 | 45 |
|  |  |  |  | 4.63 |  | -20 | -64 | 58 |
|  |  | Inferior Frontal Gyrus | 1121 | 4.90 | <0.001 | -36 | 8 | 24 |
|  |  |  |  | 4.09 |  | -36 | 5 | 38 |
|  |  |  |  | 3.82 |  | -44 | 12 | 39 |
|  |  | Medial Superior Frontal Gyrus | 521 | 4.18 | 0.002 | -4 | 23 | 38 |
|  |  |  |  | 4.09 |  | 12 | 14 | 44 |
|  |  |  |  | 3.53 |  | 2 | 14 | 44 |
|  |  |  |  |  |  |  |  |  |
| *Look* | *Valence +* | Fusiform Gyrus | 513 | 5.44 | 0.002 | 21 | -37 | -11 |
|  |  |  |  | 4.57 |  | 28 | -42 | -6 |
|  |  | Inferior Orbitofrontal Gyrus | 761 | 5.24 | <0.001 | 44 | 44 | -11 |
|  |  |  |  | 4.69 |  | 33 | 53 | -5 |
|  |  |  |  | 3.57 |  | 32 | 42 | -9 |
|  |  |  |  |  |  |  |  |  |
| *Look* | *Valence -* | Fusiform Gyrus | 3900 | 6.89 | <0.001 | -34 | -82 | -14 |
|  |  |  |  | 6.87 |  | -39 | -67 | -16 |
|  |  |  |  | 6.22 |  | -38 | -52 | -18 |
|  |  | Amygdala | 2465 | 6.46 | <0.001 | 26 | -2 | -14 |
|  |  |  |  | 5.41 |  | 28 | -10 | -10 |
|  |  |  |  | 5.10 |  | 38 | 5 | -5 |
|  |  | Fusiform Gyrus | 3507 | 6.04 | <0.001 | 39 | -74 | -14 |
|  |  |  |  | 5.80 |  | 40 | -50 | -12 |
|  |  |  |  | 5.61 |  | 45 | -66 | -14 |
|  |  | Amygdala | 2274 | 5.97 | <0.001 | -26 | -6 | -14 |
|  |  |  |  | 5.07 |  | -28 | -20 | -4 |
|  |  |  |  | 4.75 |  | -38 | -8 | -6 |
|  |  | Supramarginal Gyrus | 860 | 4.50 | <0.001 | 64 | -36 | 27 |
|  |  |  |  | 3.87 |  | 51 | -25 | 30 |
|  |  |  |  | 3.36 |  | 68 | -19 | 30 |
| Coordinates refer to MNI coordinate system. P<0.05 FWE corrected (k=10). + and - indicate a positive or negative association between brain activity and stimulus valence. | | | | | | | | |

| **Table S3. Regions implicated in emotion generation and regulation parametrically modulated by *emotional state ratings.*** | | | | | | | | |
| --- | --- | --- | --- | --- | --- | --- | --- | --- |
|  |  |  |  |  |  | **Coordinates** | | |
| **Contrast** | **Parametric regressor** | **Region** | **Size** | ***t*-value** | ***p*** | ***x*** | ***y*** | ***z*** |
| *Decrease* | *Ratings +* |  |  |  |  |  |  |  |
|  |  |  |  |  |  |  |  |  |
| *Decrease* | *Ratings -* | Inferior Frontal Gyrus | 3841 | 5.99 | <0.001 | -33 | 28 | 9 |
|  |  |  |  | 5.57 |  | -34 | -16 | -11 |
|  |  |  |  | 5.11 |  | -26 | -4 | -15 |
|  |  | Putamen | 2460 | 5.45 | <0.001 | 32 | -19 | -4 |
|  |  |  |  | 4.89 |  | 33 | -12 | -8 |
|  |  |  |  | 4.44 |  | 42 | 14 | 0 |
|  |  | Superior Parietal Lobe | 2113 | 5.03 | <0.001 | 32 | -60 | 63 |
|  |  |  |  | 4.34 |  | 36 | -52 | 56 |
|  |  |  |  | 4.29 |  | 22 | -60 | 68 |
|  |  | Inferior Occipital Gyrus | 2218 | 5.01 | <0.001 | -45 | -72 | -10 |
|  |  |  |  | 4.97 |  | -38 | -85 | -9 |
|  |  |  |  | 4.78 |  | -40 | -62 | -12 |
|  |  | Middle Temporal Gyrus | 1951 | 4.95 | <0.001 | 52 | -52 | -3 |
|  |  |  |  | 4.82 |  | 48 | -56 | -15 |
|  |  |  |  | 4.46 |  | 42 | -64 | -3 |
|  |  | Caudate | 627 | 4.94 | 0.001 | 12 | -2 | 7 |
|  |  |  |  | 3.66 |  | 6 | -19 | 15 |
|  |  |  |  | 3.38 |  | 18 | -19 | 10 |
|  |  | Supramarginal Gyrus | 775 | 4.71 | <0.001 | 64 | -32 | 44 |
|  |  |  |  | 4.11 |  | 58 | -26 | 52 |
|  |  |  |  | 3.81 |  | 52 | -28 | 45 |
|  |  |  |  |  |  |  |  |  |
| *Look* | *Ratings +* | Middle Frontal Gyrus | 1310 | 4.98 | <0.001 | 45 | 28 | 36 |
|  |  |  |  | 4.51 |  | 42 | 32 | 28 |
|  |  |  |  | 3.84 |  | 42 | 40 | 21 |
|  |  | Inferior Orbitofrontal Gyrus | 1352 | 4.94 | <0.001 | 44 | 44 | -11 |
|  |  |  |  | 4.58 |  | 40 | 50 | -2 |
|  |  |  |  | 4.35 |  | 32 | 54 | 15 |
|  |  | Cingulum | 1364 | 4.86 | <0.001 | 9 | 24 | 39 |
|  |  |  |  | 4.58 |  | 6 | 35 | 36 |
|  |  |  |  | 4.24 |  | 18 | 42 | 36 |
|  |  | Parahippocampal Gyrus | 522 | 4.69 | 0.003 | 20 | -37 | -11 |
|  |  |  |  | 4.32 |  | 26 | -43 | -9 |
|  |  | SMA | 1120 | 4.43 | <0.001 | 9 | 18 | 52 |
|  |  |  |  | 4.24 |  | 18 | 18 | 62 |
|  |  |  |  | 4.24 |  | 28 | 6 | 64 |
|  |  |  |  |  |  |  |  |  |
| *Look* | *Ratings -* | Amygdala | 4236 | 7.27 | <0.001 | 24 | -7 | -9 |
|  |  |  |  | 6.24 |  | 33 | -8 | -20 |
|  |  |  |  | 5.90 |  | 38 | 5 | -14 |
|  |  | Amygdala | 3146 | 7.04 | <0.001 | -26 | -4 | -14 |
|  |  |  |  | 5.68 |  | -39 | -6 | -11 |
|  |  |  |  | 5.49 |  | -33 | -16 | -11 |
|  |  | Supramarginal Gyrus | 1968 | 5.82 | <0.001 | 56 | -25 | 28 |
|  |  |  |  | 5.05 |  | 60 | -32 | 30 |
|  |  | Inferior Temporal Gyrus | 2926 | 5.64 | <0.001 | 44 | -43 | -11 |
|  |  |  |  | 5.55 |  | 40 | -76 | -12 |
|  |  |  |  | 4.77 |  | 45 | -55 | 1 |
|  |  | Thalamus | 450 | 5.33 | 0.005 | 0 | -7 | 8 |
|  |  |  |  | 4.30 |  | -2 | -16 | 14 |
|  |  | Inferior Occipital Gyrus | 2116 | 5.14 | <0.001 | -39 | -80 | -11 |
|  |  |  |  | 4.64 |  | -45 | -46 | -12 |
|  |  |  |  | 4.51 |  | -39 | -67 | -4 |
|  |  |  |  |  |  |  |  |  |
|  |  | Supramarginal Gyrus | 507 | 4.79 | 0.003 | -58 | -25 | 25 |
|  |  | Anterior Cingulate | 606 | 4.75 | 0.002 | -2 | 23 | 26 |
|  |  |  |  | 3.90 |  | 4 | 12 | 38 |
|  |  |  |  | 3.42 |  | 2 | 5 | 34 |
|  |  | Hippocampus | 829 | 4.58 | <0.001 | -12 | -26 | -9 |
|  |  |  |  | 4.33 |  | -4 | -37 | -18 |
|  |  |  |  | 4.00 |  | -9 | -30 | -16 |
| Coordinates refer to MNI coordinate system. P<0.05 FWE corrected (k=10). + and - indicate a positive or negative association between brain activity and emotional state ratings. | | | | | | | | |
